# Intravesical instillation-based mTOR-STAT3 dual targeting for bladder cancer treatment

**DOI:** 10.1186/s13046-024-03088-7

**Published:** 2024-06-18

**Authors:** Dae Hoon Lee, Jung Ki Yoo, Ki Hwan Um, Wootae Ha, Soo Min Lee, Junseong Park, Min Jeong Kye, Jungyo Suh, Jin Woo Choi

**Affiliations:** 1 Department of Pharmacology, College of Pharmacy, Kyung Hee University, Seoul, 02447 Republic of Korea; 2 Department of Biomedical and Pharmaceutical Sciences, College of Pharmacy, Kyung Hee University, Seoul, 02447 Republic of Korea; 3 R&D Center of Curigin Ltd., Curigin, Seoul, 04778 Republic of Korea; 4 Department of Regulatory Science, College of Pharmacy, Kyung Hee University, Seoul, 02447 Republic of Korea; 5 Precision Medicine Research Center, College of Medicine, The Catholic University of Korea, Seoul, 06591 Republic of Korea; 6 Department of Urology, Asan Medical Center, University of Ulsan College of Medicine, Seoul, 05505 Republic of Korea

**Keywords:** Bladder cancer, mTOR, STAT3, siRNA, Cancer therapy

## Abstract

**Background:**

Recent intravesical administration of adenoviral vectors, either as a single injection or in combination with immune checkpoint inhibitors, exemplified by cretostimogene grenadenorepvec and nadofaragene firadenovec, has demonstrated remarkable efficacy in clinical trials for non-muscle invasive bladder cancer. Despite their ability to induce an enhanced immune reaction within the lesion, the intracellular survival signaling of cancer cells has not been thoroughly addressed.

**Methods:**

An analysis of the prognostic data revealed a high probability of therapeutic efficacy with simultaneous inhibition of mTOR and STAT3. Considering the challenges of limited pharmaco-accessibility to the bladder due to its pathophysiological structure and the partially undruggable nature of target molecules, we designed a dual siRNA system targeting both mRNAs. Subsequently, this dual siRNA system was encoded into the adenovirus 5/3 (Ad 5/3) to enhance in vivo delivery efficiency.

**Results:**

Gene-targeting efficacy was assessed using cells isolated from xenografted tumors using a single-cell analysis system. Our strategy demonstrated a balanced downregulation of mTOR and STAT3 at the single-cell resolution, both in vitro and in vivo. This approach reduced tumor growth in bladder cancer xenograft and orthotopic animal experiments. In addition, increased infiltration of CD8^+^ T cells was observed in a humanized mouse model. We provided helpful and safe tissue distribution data for intravesical therapy of siRNAs coding adenoviruses.

**Conclusions:**

The bi-specific siRNA strategy, encapsulated in an adenovirus, could be a promising tool to augment cancer treatment efficacy and overcome conventional therapy limitations associated with “undruggability.” Hence, we propose that dual targeting of mTOR and STAT3 is an advantageous strategy for intravesical therapy using adenoviruses.

**Graphical Abstract:**

The current investigation introduces an innovative conceptualization of bispecific short hairpin RNA (bs_shRNA) tailored for the equilibrated modulation of dual genes within a singular cellular context. This novel bs_shRNA was loaded into the genome of an oncolytic adenovirus to augment the therapeutic efficacy of oncolytic viral interventions via the targeted inhibition of mTOR and STAT3 pathways. In addition, the administration of BSV significantly reduced the volume of bladder cancer tumors, concomitantly facilitating an enhanced recruitment of CD8^+^T lymphocytes in vivo. 
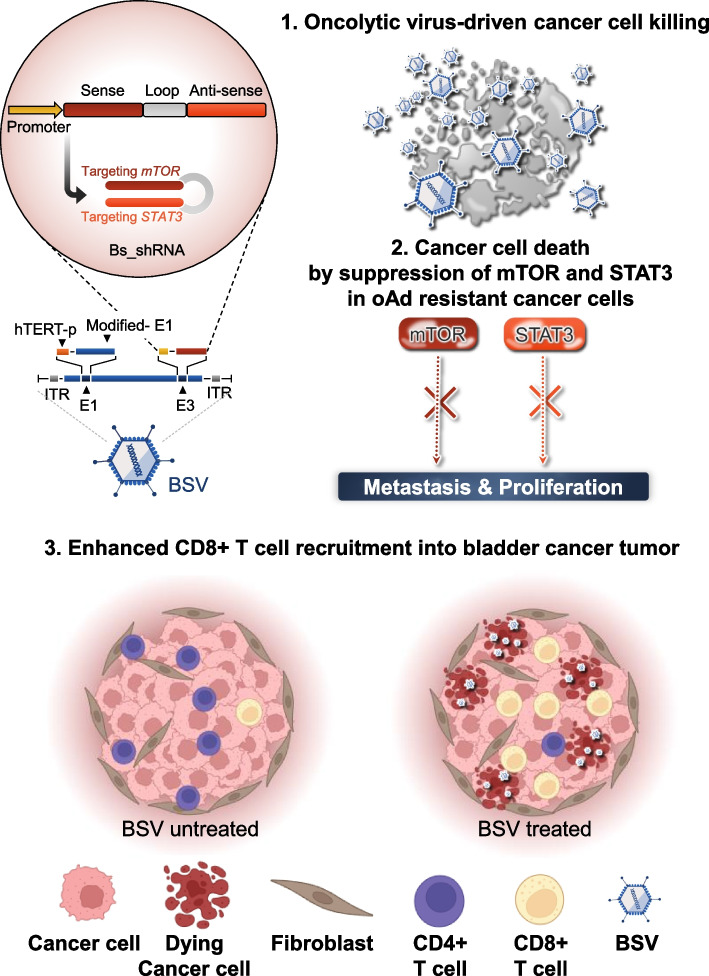

**Supplementary Information:**

The online version contains supplementary material available at 10.1186/s13046-024-03088-7.

## Background

Bladder cancer is one of the most lethal urological malignancies, the treatment of which has not witnessed a significant overhaul since the 1970s. Bacillus Calmette-Guerin (BCG)-based therapy has remained the standard treatment protocol [1]. Although the use of a combination of pembrolizumab and gemcitabine for BCG-unresponsive non-muscle invasive bladder cancer (NMIBC) has been recently approved [2], targeted therapies remain sparse relative to the advancements in other cancer types [3]. This could be partially attributed to the histological characteristics of the bladder, such as its limited vasculature [4]. Recent advancements have facilitated the efficacious delivery of therapeutic agents into lesions by employing cretostimogene grenadenorepvec (CG0070), an adenoviral vector encoding granulocyte–macrophage colony-stimulating factor (GM-CSF). Its administration via urethral instillation instigates immunosurveillance and has demonstrated encouraging results in Phase III clinical trials [5]. Concurrently, nadofaragene firadenovec-vncg (Adstiladrin™) represents an alternative approach to gene therapy. In this methodology, a replication-deficient adenovirus is skillfully engineered to secrete interferon-α, thereby stimulating the immune system [6]. Despite utilizing adenoviruses for patients with BCG-unresponsive NMIBC with no other treatment options, the bladder’s unique encapsulated system is conducive to successful viral gene therapy, with an acceptable safety profile. However, challenges persist in optimizing delivery methods for viral gene therapy. Although intratumoral injection, as observed with talimogene laherparepvec (Imlygic™), has been the primary strategy for tumor targeting, outcomes could vary based on factors such as operator technique and tumor cell absorption [7]. Thus, limited distribution to closed tissues such as those of the bladder suggests that alternative delivery routes such as intravesical instillation could offer safer and more effective gene therapy options.

The therapeutic efficacy of currently available targeted treatments and chemotherapies is constrained in numerous malignancies, consequently leading to less-than-desirable response rates. In addition, resistance due to genetic mutations or other drug-desensitizing mechanisms of cancer cells [8] often trails single-gene targeting protocols [9]. Given the intrinsic genetic heterogeneity of cancer, there is an impelling necessity to design another therapeutic strategy to effectively target multiple oncogenic driver genes, thus amplifying the potency of the treatment and potentially improving patient survival [10, 11]. Although combinatory regimens are considered for clinical benefits, unfortunately over 85% of idealistic targets, expected to have better therapeutic efficacy, have an undruggable property with current modalities [12].

Recently, RNA interference (RNAi) has gained considerable attention as a potentially transformative therapeutic approach for cancer treatment [13]. Considering its mode of action that targets mRNA rather than proteins, this strategy could prove beneficial to shift the focus toward undruggable targets, a realm distinct from other therapeutic modalities such as chemical compounds and antibodies.

Numerous genes remain classified as undruggable, including but not limited to *c-myc* and *k-ras*. Notably, signal transducer and activator of transcription 3 (STAT3) are included in this category. Current mTOR inhibitors predominantly target mTORC1, leaving mTORC2 largely undruggable, and their utilization is constrained due to adverse effects [14, 15]. The mTOR signaling pathway has been implicated in orchestrating cell growth, proliferation, and survival [16]. Conversely, STAT3 has been correlated with cancer cell survival, angiogenesis, and evasion of immune surveillance [17]. However, despite their central importance, mTOR and STAT3 pose formidable challenges in terms of therapeutic targeting. Targeting STAT3 has proved to be arduous due to unforeseen downstream ramifications and its non-enzymatic properties [18, 19]. Considering these limitations, the current study presents a pioneering bispecific short hairpin RNA (shRNA) system meticulously designed to concurrently downregulate two key oncogenic genes, namely, *mTOR* and *STAT3*. We encoded the sequence to be transcribed into shRNA within oncolytic adenoviral vectors to ensure efficacious delivery of this bispecific siRNA into cancer cells.

In the subsequent portion of this manuscript, we direct our therapeutic efforts toward bladder cancer. Furthermore, we attempted bladder cancer treatment with our bi-specific siRNA coding adenovirus system to improve the established safety of previous gene therapies. Our initial target gene pair, *mTOR* and *STAT3*, is highly significant in bladder cancer. This study unveils a distinctive bispecific siRNA tool to concurrently downregulate multiple survival genes within individual cells. The potential applications of this tool extend beyond the realm of bladder cancer to encompass a multitude of other undruggable genes and fatal diseases for both viral and non-viral delivery systems.

## Methods

### Survival data analysis

The RNA sequencing data of clinical records were used in the public repository from cBioportal (Pancancer Atlas, TCGA; 2018). It included data from 407 patients from the bladder urothelial carcinoma cohort and 487 patients from the lung squamous cell carcinoma cohort (PanCancer Atlas, TCGA). Kaplan–Meier survival analysis was performed to compare patient survival according to the *mTOR* and *STAT3* gene expression. Log-rank test was used to determine the statistical difference in overall survival between groups. All statistical analyses were performed using R 4.0.2 (survival package 3.2–12 and survminer R package 0.4.9). The significance of human data analysis was evaluated using the two-tailed Log-rank test. All in vitro experiments statistical tests were two-tailed student’s *t*-tests, and *p*-values less than 0.05 were considered statistically significant.

### Cell lines and chemicals

The 253 J-BV and RT-4 human bladder cancer cell lines, C4-2B human prostate carcinoma cell line, PrEC human primary prostate epithelial cell line, HeLa uterus adenocarcinoma cell line, and HEK293 human embryonic kidney cell line were purchased from the American Type Culture Collection (ATCC, Virginia, US). A549 lung cancer cell line was purchased from the Korean Cell Line Bank (KCLB, Seoul, Korea). The human urethra epithelial cell (HUEpC) line was purchased from Cell Applications, Inc. (California, US). Briefly, 253 J-BV, RT-4, HeLa, and HEK293 cells were cultured in Dulbecco’s modified Eagle’s medium (DMEM, Cat. no. 11965–092, Thermo Fisher Scientific Inc., Massachusetts, US). C4-2B and A549 cell lines were cultured in RPMI 1640 (Cat. no. 11875–093, Thermo Fisher Scientific Inc.). Both DMEM and RPMI1640 were supplemented with 10% fetal bovine serum (FBS, Cat. no. 26140079; Gibco Inc., Texas, US) and 1% penicillin–streptomycin–glutamine 100 × (Cat. no. 15140122; Gibco Inc., Texas, US). PrEC cells were cultured in PrEGM (Cat. no. CC-3166, Lonza, Basel, Swiss). HUEpC cells were cultured in the HUEpC growth medium (Cell Applications, Inc., California, US). Cells were grown at 37 °C and 5% CO_2_. The Torin-1 mTOR inhibitor (Cat. no. 14379 s; Cell Signaling Inc., Massachusetts, US) and STATTIC STAT3 inhibitor (Cat. no. 97598 s; Cell Signaling Inc.) were diluted in dimethyl sulfoxide (DMSO, Cat. no. D2650; Sigma Inc., Mijuri, US) for treatment. Only the surviving cells were collected in a growth medium with 1.5 µg/ml cisplatin and grown to establish cisplatin-resistant 253 J-BV cells.

### Real-time PCR analysis (qPCR)

The RNA was extracted from each sample using TRIzol (Cat. no. 15596018; Thermo Fisher Scientific Inc., Massachusetts, US).After mRNA extraction, the cDNA was synthesized from RNA using a High-Capacity cDNA Reverse Transcription Kit (Cat. no. 4368814; Thermo Fisher Scientific Inc.). Real-time polymerase chain reaction (PCR) was conducted using the QuantStudio™ 3 Real-Time PCR System (Cat. no. A28567; Thermo Fisher Scientific Inc.). Every experiment was performed more than thrice individually, and statistical significance was calculated by *t*-test.

### Cell viability assay

We calculated the ratio of live and dead cells after harvesting cells by staining them with trypan blue (Cat. no. 15250061; Thermo Fisher Scientific Inc.,) and assessed using Countess 3 (Cat. no. A50298; Thermo Fisher Scientific Inc) to automatically measure the number of live and dead cells. Every counting was performed thrice for each batch. To assess the cell viability, these were fixed with chilled methanol for 5 min at − 20℃, followed by staining with 1% crystal violet solution and washing thrice with phosphate-buffered saline (PBS; Cat. no. 10010–049; Gibco Inc., Texas, US).

### Western blotting

Virus-infected cells were collected in 1 × PBS by centrifugation at 300 × *g* for 5 min and subsequently lysed using the Cell Lysis Buffer 10 × (Cat. no. 9803 s; Cell Signaling Inc., Massachusetts, US) diluted with distilled water (1:10) for 15 min on ice. Cell lysates were centrifuged at 12 000 × *g* for 10 min to harvest the supernatant. Supernatants were mixed with 5 × sodium dodecyl sulfate–polyacrylamide gel electrophoresis (SDS-PAGE) loading buffer (Cat. no. CBS002; LPS Solution Inc., Daejeon, Korea) and heated at 100 °C for 10 min. The samples were loaded in SDS-PAGE and electrophoresed at 80 and 120 V for 30 min and 90 min, respectively. Proteins in the SDS-PAGE were transferred to polyvinylidene fluoride (PVDF) membranes (Cat. no. HVLP02500; Merck Inc., New Jersey, US). Membranes were incubated in 5% skim milk in TBS-T buffer (Cat.no. CBT007L; LPS Solution Inc., Daejeon, Korea) for 60 min. Subsequently, the membranes were incubated in primary antibodies at 4 °C overnight. Antibodies used included phosphorylated mTOR (Cat. no. 2971 s; Cell Signaling Inc., Massachusetts, US), mTOR (Cat. no. 2972 s, Cell Signaling Inc.), phosphorylated STAT3 (Cat. no. 9139 s, Cell Signaling Inc.), STAT3 (Cat. no. 9145 s, Cell Signaling Inc.), phosphorylated 4E-BP1 (Cat. no. 2855 s, Cell Signaling Inc.), 4E-BP1 (Cat. no. 9644 s, Cell Signaling Inc.), phosphorylated p70S6K (T389) (Cat. no. 9205 s, Cell Signaling Inc.), p70S6K (Cat. no. 9202 s, Cell Signaling Inc.), phosphorylated Akt (Cat. no. 9271 s, Cell Signaling Inc.), Akt (Cat. no. 9272 s, Cell Signaling Inc.), phosphorylated FAK (Cat. no. 3283 s, Cell Signaling Inc.), FAK (Cat. no. 3285 s, Cell Signaling Inc.), and β-actin (Cat. no. sc-47778; Santa Cruz Inc., California, US). Following washes in TBS-T buffer, membranes were incubated with 1:10,000 anti-mouse horse radish peroxidase (HRP; Cat.no. sc-2005; Santa Cruz Inc.) and anti-rabbit HRP antibody (Cat. no. sc-2357; Santa Cruz Inc.) in TBS at 25 °C for 1 h. The membranes were detected using an iBright western blot imaging system (Cat. no. CL1000, Thermo Fisher Scientific Inc.).

### Copy number calculation of shRNA

The copy number of shRNA per cell was calculated by transfecting 80% confluent 253 J-BV with 2 µg of pAd1129 vectors coding t_shRNA (pAd1129-t_shRNA) and bs_shRNA (pAd1129-bs_shRNA) on 6-well plates. After 48 h, the cells were harvested with TRIzol and both siRNAs of mTOR and STAT3 were processed to cDNAs using the miScript II RT kit (Cat. no. 218161; QIAGEN Inc., Venlo, Netherlands). cDNA number was calculated with real-time PCR based on the CT value-copy number standard curve.

### RNA preparation and qPCR from a single cell

We evaluated the mRNA expression at a single cell level by transfecting 80% confluent 253 J-BV with 2 µg of pAd1129 vectors coding t_shRNA (RNA sequence: GAC UGU GGC AUC CAC CUG CAU = UU/GAC UGA GGC GCC UAC CUG CAU = UU and AUG CAG GUG GAU GCC ACA GUC = UU/AUG CAG GUA GGC GCC UCA GUC = UU) (pAd1129-t_shRNA) and bs_shRNA (RNA sequence: GAC UGU GGC AUC CAC CUG CAU = UU/AUG CAG GUA GGC GCC UCA GUC = UU) (pAd1129-bs_shRNA) on 6-well plates. After 48 h, cells were collected as single cell units (1 µL volume each) using the Micro Pick and Place System (Nepa Gene Co., Ltd., Ichikawa, Japan). Subsequently, the mRNA was extracted from each single cell using the single-cell MicroGEM RNAGEM RNA Prep Kit (Cat. no. RTP0500; MicroGEM Inc., Virginia, US).cDNA was synthesized using the HiSenScript RH(-) cDNA Synthesis Kit (Cat. no. 25014; iNtRON Inc., Gyeonggi, Korea). Real-time PCR analysis was performed to confirm the expression.

### Preparation of replication-competent adenovirus

The bispecific shRNA-expressing virus (BSV) was generated from cosmids, which were constructed from plasmid adenoviral vectors from O.D. 260 Inc. (Idaho, US; pAd1127 vector, cat. no. QP-04 for *E1* and *pIX* genes; pAd1128 vector, cat. no. QP-09 for *E2* and late genes; pAd1129, cat. no. QP-10, for *E3* and fiber genes; and pAd1130, cat. no. QP-13 for *E4* genes). In particular, BSV was modified to be replicated selectively on cancer cells. Briefly, the human telomerase reverse transcriptase (hTERT) promotor was inserted at the Spe1 restriction enzyme site before the E1 region in the pAd1127 vector. The bispecific shRNA was produced by inserting the U6 promoter in front of the E3 gene region, followed by the bispecific shRNA sequence (GACTGTGGCATCCACCTGCATTTGGATCCAAATGCAGGTAGGCGCCTCAGTCTT). The cosmid vector was established using a combination of four modified vectors, namely, pAd1127, pAd1128, pAd1129, and pAd1130. After digestion with *PacI* (Cat. no. ER2201; Thermo Fisher Scientific Inc.), the cosmid was transfected into 293A cells (Cat. no. R70507; Invitrogen, Massachusetts, US) using the CalPhos mammalian transfection kit (Cat. no. 631312; Clontech, California, US) to harvest the master viral seed stock. The virus was purified using the Adeno-X mega purification kit (Cat. no. 631032; Clontech). For titration, the virus was used to infect HEK293 cells (Cat. no. CRL-1573TM; ATCC, US) and tittered using the Adeno-X rapid titer kit (Cat. no. 632250; Clontech) to calculate the infectious units (IFUs). The viral number used for subsequent experiments was measured as IFU units.

### RNA sequencing and data analysis

The total RNA was isolated using the TRIzol reagent (Cat. no. 15596018; Thermo Fisher Scientific Inc.). The RNA quality was assessed using an Agilent 2100 bioanalyzer (Agilent Technologies, Amstelveen, The Netherlands), whereas it was quantified using an ND-2000 Spectrophotometer (Thermo Fisher Scientific Inc.).

Libraries were constructed from the total RNA using the NEBNext Ultra II Directional RNA-Seq Kit (New England BioLabs Inc., Massachusetts, US). The mRNA was isolated using the Poly(A) RNA Selection Kit (Lexogen Inc., Vienna, Austria). Isolated mRNAs were used for cDNA synthesis and shearing following the manufacturer’s instructions. Indexing was performed using the Illumina indexes 1–12. The enrichment step was conducted using PCR. Subsequently, the libraries were checked using the Agilent 2100 bioanalyzer (DNA High Sensitivity Kit) to evaluate the mean fragment size. Quantification was performed using the library quantification kit in a StepOne Real-Time PCR System (Life Technologies Inc., California, US). High-throughput sequencing was performed as paired-end 100 sequencing using NovaSeq 6000 (Illumina Inc., California, US).

Quality control of raw sequencing data was performed using FastQC [20, 21]. Adapter and low-quality reads (< Q20) were removed using FASTX_Trimmer (https://hannonlab.schl.edu/fastx_toolkit/) and BBMap (https://www.geneious.com/plugins/bbm). Trimmed reads were mapped to the reference genome using TopHat (https://ccb.jhu.edu/software/tophat/). The gene expression was estimated using fragments per kb per million reads (FPKM) values by Cufflinks (https://cole-trapnell-lab.github.io/cufflinks/). The FPKM values were normalized according to the quantile normalization method using EdgeR within R [22, 23]. Data mining and graphic visualization were performed using ExDEGA (Ebiogen Inc., Seoul, Korea).

#### Single-cell RNA seq and data processing

The 253 J-BV cells were treated with 5 MOI CV and BSV and subsequently collected. The BD Rhapsody single-cell analysis system (BD biosciences Inc., New Jersey US) was used to capture every cell using barcorded beads and lysed for hybridization of mRNA. After retrieving the beads, the cDNA was synthesized and gene expression was analyzed. All procedures were performed following the BD Rhapsody protocol. The BD Rhapsody assays were used to generate sequencing libraries using single-cell multiomic experiments. The analysis pipeline works with paired-end FASTQ Read 1 and Read 2 files generated from Illumina sequencers. The minimum read length required was 60 bp for Read 1 and 42 bp for Read 2. Read 1 contained information on cell labels and molecular identifiers, whereas Read 2 contained information on the gene. After initial processing, the scRNA-seq data of 37,173 cells with 114,346,006,622 total reads were generated. Subsequent analyses, including normalization (SCTransform), unsupervised clustering, UMAP dimensionality reduction, and DEG analyses, were conducted using the R package Seurat (v4.0.6) to identify, characterize, and visualize clusters [24]. Pseudotime trajectory analysis was performed using the R package Monocle3 [25]. The Seurat object was transformed to a cell_data_set and was subsequently supplied as an input to Monocle3. Pseudotime trajectories were constructed using UMAP embeddings deducted from Seurat, with the C2 cluster as the root of the trajectories.

#### Animal Experiments

Five-week-old BALB/c nude male (only for Fig. 8A-C) and female mice were purchased from Orient Bio (Gyeonggi, Korea). All animal experiments were reviewed and approved by the Institutional Animal Care and Use Committee (IACUC, approval number: CRG-RNDC02.01–02) and performed according to the criteria of the guidelines of IACUC. Mice were maintained in pathogen-free facilities.

We established the subcutaneous tumor xenograft mouse model; 1 × 10^6^ cells were subcutaneously injected into the right flanks of mice. For the ex vivo experiment, 253 J-BV cells were infected with the virus 1 h before their injection into mice. The multiplicity of infection (MOI) was 2 for 253 J-BV. For the in vivo intratumoral injection, non-infected cells (1 × 10^6^) were subcutaneously injected into mice.

When the tumor reached a specific size, 1 × 10^8^ or 1 × 10^9^ IFUs of control virus (CV) and bispecific shRNA-expressing virus (BSV) were injected intratumorally or 1 × 10^8^ IFUs of CV and BSV were intratumorally injected as either a single or multiple shots after randomizing mice to remove bias. An individual who was not related to this project conducted drug treatment only to remove bias. For cisplatin combination therapy, 1 × 10^8^ IFUs of BSV were intratumorally injected on days 1 to 3 daily and intraperitoneally(10 μM) on days 1 to 10.

For Fig. 7C–G, all mice received a single shot intratumoral injection and were sacrificed at days 0, 1, 3, 7, and 14. Their tumors and organs were harvested for real-time PCR and immunofluorescence staining. Immune cell infiltration into tumors was analyzed (Fig. 9) using six CD34^+^ humanized mice (CD34 + hu-NSG; The Jackson Laboratory, Maine, USA) per each group following the previously established xenograft mouse protocol of 253 J-BV.

#### Immunofluorescence

Tissues were sliced with optimal cutting temperature (OCT) compound using a cryostat (Cat. no. CM1800; Leica, Wetzlar, Germany). After sectioning, the samples were initially stained with hexon (Cat. no. MA1-7328, Invitrogen), cleaved caspase-3 (Cat. no. 9661 s, Cell Signaling Inc.), CD31 (Cat. no. 3528 s, Cell Signaling Inc.), vimentin (Cat. no. 9856 s, Cell Signaling Inc.), CD3 (Cat. no. MABF413; Merck, New Jersey, US), CD4 (Cat. no. 58–0042-82; Invitrogen, Massachusetts, US), CD8 (Cat. no. MHCD0826; Invitrogen,), and smooth muscle actin (Cat. no. ab7817; Abcam, Cambridge, UK) antibodies for 1 h. The samples were incubated with primary antibodies diluted in 1% bovine serum albumin (BSA) in PBST overnight in a humidified chamber at 4 °C. After incubation with primary antibodies, the samples were washed thrice with PBS for 5 min per washing. Afterward, the samples were incubated with secondary antibodies (Cat. no, ab150077 and ab150078, Abcam; Cat. no. 8890 s, 4412 s; Cell Signaling Inc.), followed by incubation with a secondary antibody diluted in 1% BSA in PBST for 1 h in the dark at 25 °C for fluorescence staining.To remove background signals, samples were washed thrice using PBS for 5 min, each in the dark.

#### Orthotopic bladder *cancer* model

We established the orthotopic bladder cancer mouse model by injecting 253 J-BV-luc cells into the mouse bladder vesicle through intravesical instillation.5 × 10^6^ cells were dissolved in 30 µl of 5% Matrigel dissolved in PBS and injected into each entity. Seven days later (day 1), viruses were injected into the bladder vesical through instillation. Multiple injections were administered on day 2 (for double- and triple-injected groups) and day 3 (for triple-injected groups). For bioluminescence imaging, tumor growths were checked using VISQUE SMART-LF (Cat.no. BI24001; Vieworks, Gyeonggi-do, Korea).

#### Biodistribution analysis for intravesical BSV instillation

A total of 4.8 × 10^10^ IFUs/kg of BSV were administered via intravesical instillation in the hamster model. At each designated time point after administration, the animals within each group were humanely sacrificed, and their organs were subsequently analyzed for viral distribution. The genomic DNA from each organ sample was extracted, and the number of viral particles was quantified based on viral genome copy number. This study was conducted using a sample size of n = 5 hamsters per group, each of which was aged 8 weeks old (Orient Bio, Gyeonggi, Korea). This study was conducted in accordance with the Good Laboratory Practice (GLP) standards.

#### Fluorescence-activated cell sorter (flow cytometry)

Tissue samples were collected and dissociated into single cell level using 2 mg/mL of collagenase type 1 (Cat. no. 17018029; Thermo Fisher Scientific Inc.), 1 mg/mL of hyaluronidase type 2 (Cat. no. H2126; Sigma Inc., Mijuri, US). The dissociated single cells were immunostained using antibodies tagged with fluorescent dye to study protein expression. Briefly, CD3 (Cat. no. MABF413; Merck, New Jersey, US), CD4 (Cat. no. 58–0042-82; Invitrogen, Massachusetts, US), CD8 (Cat. no. MHCD0826; Invitrogen,), and CD45 (ab200315; Abcam) were used for protein labeling. After labeling, the samples were analyzed using the CytoFLEX Flow cytometer (Cat. no. C02945; Beckman, California, US).

## Results

### mTOR and STAT3: a synergistic alliance in patients with bladder *cancer*

**Fig. 1:**
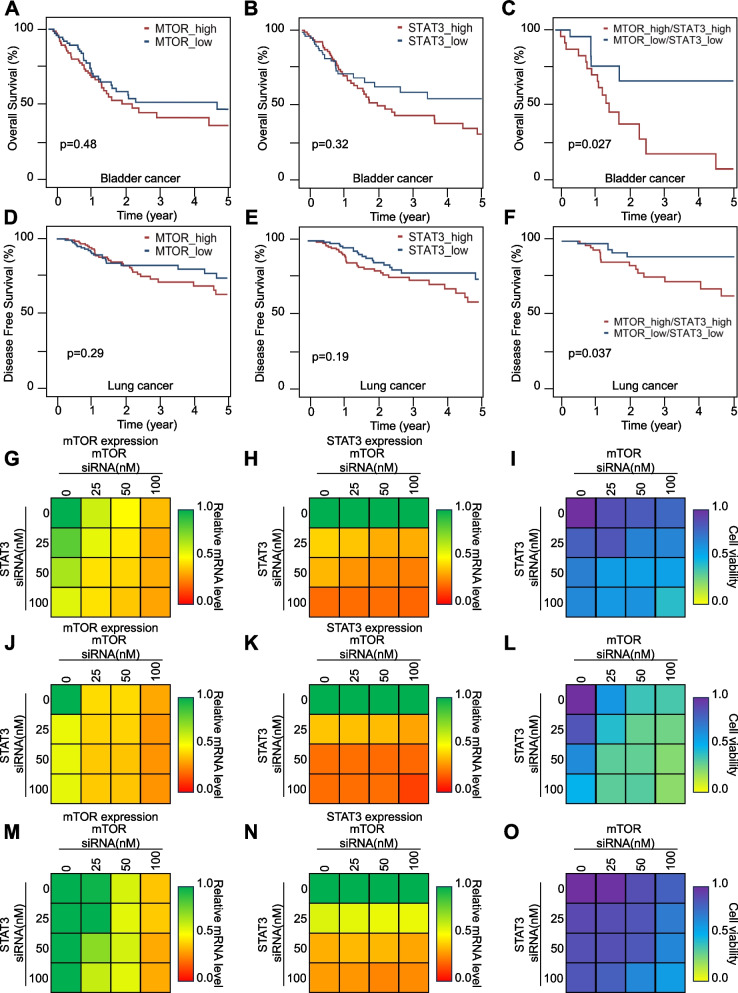
mTOR and STAT3: a synergistic alliance in patients with bladder cancer. **A-B** Overall survival in patients with bladder cancer. Among 407 bladder cancer samples, patients with an upper 20% (79 patients) and lower 20% (79 patients) expression of mTOR (**A**) or patients with an upper 20% (81 patients) and lower 20% (81 patients) expression of STAT3 (**B**) were selected and their survival rate was analyzed using the Kaplan–Meyers curve. **C** Survival of patients with commonly high (24 patients) or low (26 patients) expression coindex of mTOR and STAT3. **D**-**E** Disease-free survival (DFS) in patients with lung squamous cell carcinoma (LUSC). Among 487 LUSC samples, patients with an upper 20% (77 patients) and lower 20% (65 patients) expression of mTOR (**D**) or patients with an upper 20% (71 patients) and lower 20% (68 patients) expression of STAT3 (**E**) were selected and their survival rate was analyzed in a Kaplan-Meyers curve. **F** DFS of patients with commonly high (27 patients) or low (23 patients) expression coindex of mTOR and STAT3. Refer to survival data analysis in methods for detail method about (**A**-**F**). **G**-**O** The transfection of si-mTOR and si-STAT3 was performed in A549 cells (**G**-**I**), C4-2B cells (**J**-**L**), and 253 J-BV cells (**M**–**O**) in strict accordance with the concentration defined in the associated plot. A PCR assay was used to evaluate the mRNA expression of mTOR (G, **J**, and **M**) and STAT3 (**H**, **K**, and **N**). Finally, cell viability was analyzed using an appropriate cell viability assay (**I**, **L**, and **O**). To ensure uniformity in the total RNA content across all samples, a negative siRNA control was introduced to each, up to a concentration of 200 nM. Subsequently, all samples were collected for analysis 48 h post-transfection

The patient survival data analysis results predicted mTOR and STAT3 as the most associated with bladder cancer. Gene expression data and survival information for 407 patients with bladder cancer were amassed from the TCGA database. We observed that neither mTOR nor STAT3 alone served as a key determinant in patient prognosis (Fig. 1A, B). However, the combined evaluation of these two genes suggested that these could be used as a critical gene set for predicting patient outcomes (Fig. 1C). Similarly, neither mTOR nor STAT3 alone served as a key determinant in lung cancer patient prognosis (Fig. 1D, E). But the combined evaluation of mTOR and STAT3 genes suggested that these also could be used as a critical gene set for predicting lung cancer patient outcomes (Fig. 1F).

We next assessed whether their synergistic effect was reproduced in vitro and what concentration was the most effective. For this, we transfected the A549 (Fig. 1G–I), C42B (Fig. 1J-L) and 253 J-BV cells (Fig. 1M–O) with a combination of siRNAs targeting mTOR and STAT3 in a concentration-dependent manner. The transfection of si-mTOR resulted in a dose-dependent downregulation of mTOR expression, as evidenced by Fig. 1G, J, and M. Additionally, it appears that mTOR expression was affected by si-STAT3 transfection. Conversely, the expression of STAT3 was dose-dependently downregulated following si-STAT3 transfection, as depicted in Fig. 1H, K, and N. However, STAT3 expression remained unaffected by si-mTOR transfection. By the results of gene expression and cell viability, we found that the synergy effect was considerably more significant using this combination. In this setting, si-mTOR exerted a stronger cell death effect. The 50 nM concentration of siSTAT3 strongly supported mTOR suppression-induced cell death. The additive effect displayed a gradual increase (Fig. 1I, L, and O).

### Efficacy of combined suppression between mTOR and STAT3

**Fig. 2:**
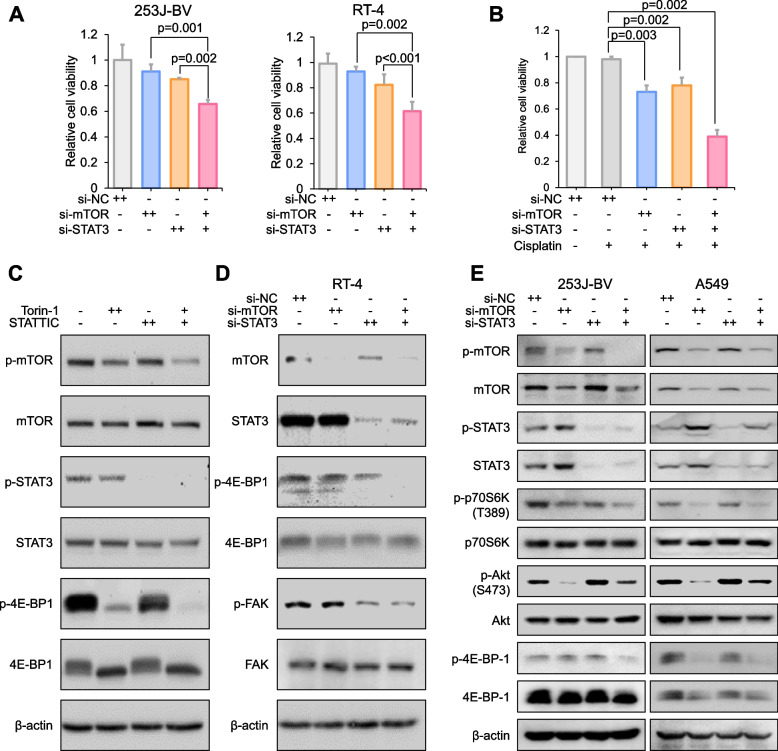
Efficacy of combined suppression between mTOR and STAT3. **A** To evaluate the synergistic effect, the viabilities of 253 J-BV and RT-4 cells were measured following individual or combined treatment with mTOR and STAT3 siRNAs (+ : siRNA 50 nM, +  + : siRNA 100 nM). **B** Viability of cisplatin-resistant 253 J-BV cells in the presence of cisplatin (10 μM) with siRNA treatment (+ : siRNA 50 nM, +  + : siRNA 100 nM). **C** Western blotting to evaluate the downstream targets of mTOR and STAT3. Downstream target molecules were evaluated after treatment with Torin1 and STATTIC in 253 J-BV cells (Torin-1; + : 1 μM, +  + : 2 μM, STATTIC; + : 5 μM, +  + : 10 μM). **D** RT-4 cells were transfected with mTOR and STAT3 siRNAs. Next, mTOR- and STAT3-related molecules were analyzed (+ : siRNA 50 nM, +  + : siRNA 100 nM). **E** The 253 J-BV and A549 cell lines were subjected to transfection procedures with siRNAs targeted at mTOR and STAT3. Subsequent to this manipulation, a detailed analysis was conducted focusing on molecular entities related to both mTORC1 and mTORC2 complexes (+ : siRNA 50 nM, +  + : siRNA 100 nM) (for statistics, two-tailed *t*-test for **A** and **B**)

Subsequently, we examined whether the mTOR and STAT3 double knockdown would prove more advantageous than single gene knockdowns of each gene as a therapeutic strategy in bladder cancer. The mTOR/STAT3 double knock-down diminished cell viability in two distinct bladder cancer cell lines at half the concentration of each siRNA compared to single targeting of each gene (Fig. 2A). Cisplatin is administered as the primary chemotherapeutic agent to treat patients with muscle-invasive bladder cancer; however, cancer cells can develop drug resistance within a few months [26]. To investigate the synergistic effect of mTOR/STAT3 siRNA with cisplatin, we established a cisplatin-resistant cell line and subsequently treated it with mTOR and STAT3 siRNAs. We found that co-treatment with mTOR and STAT3 siRNAs significantly enhanced cell death of cisplatin-resistant 253 J-BV cells by approximately 70% (Fig. 2B). Next, 253 J-BV were treated with Torin-1 and STATTIC, inhibitors of mTOR and STAT3, respectively, to discern any alterations in the downstream signaling pathway. We analyzed mTOR- and STAT3-related molecules via western blotting. Pharmacological inhibition of both pathways exhibited a synergistic effect on phospho-4E-BP1 levels, a key mediator of proliferation and the mTOR signaling pathway (Fig. 2C). Intriguingly, these findings were replicated in cells treated with half the concentration of mTOR and STAT3 siRNAs (Fig. 2D). The focal adhesion kinase (FAK), a consequential downstream constituent of STAT3, exhibited a marked presence in invasive cancer cells, exemplified by RT-4 in our experimental condition, was studied to explore the potential synergistic effects emanating from the perspective of the STAT3 pathway. The siRNA-mediated mTOR and STAT3 effectively inhibited the phosphorylation of FAK (second and third blots from the bottom in Fig. 2D). We checked the phosphorylation of S6K1 and AKT as indicators for the activities of mTORC1 and mTORC2, respectively. The phosphorylation at S6K1 T389 and AKT S473 sites reduce following the treatment with si-mTOR and, even by the low dose combination of si-mTOR and si-STAT3 (Fig. 2E).

### Balanced knockdown strategy for mTOR/STAT3 via the shRNA-mediated expression system

**Fig. 3:**
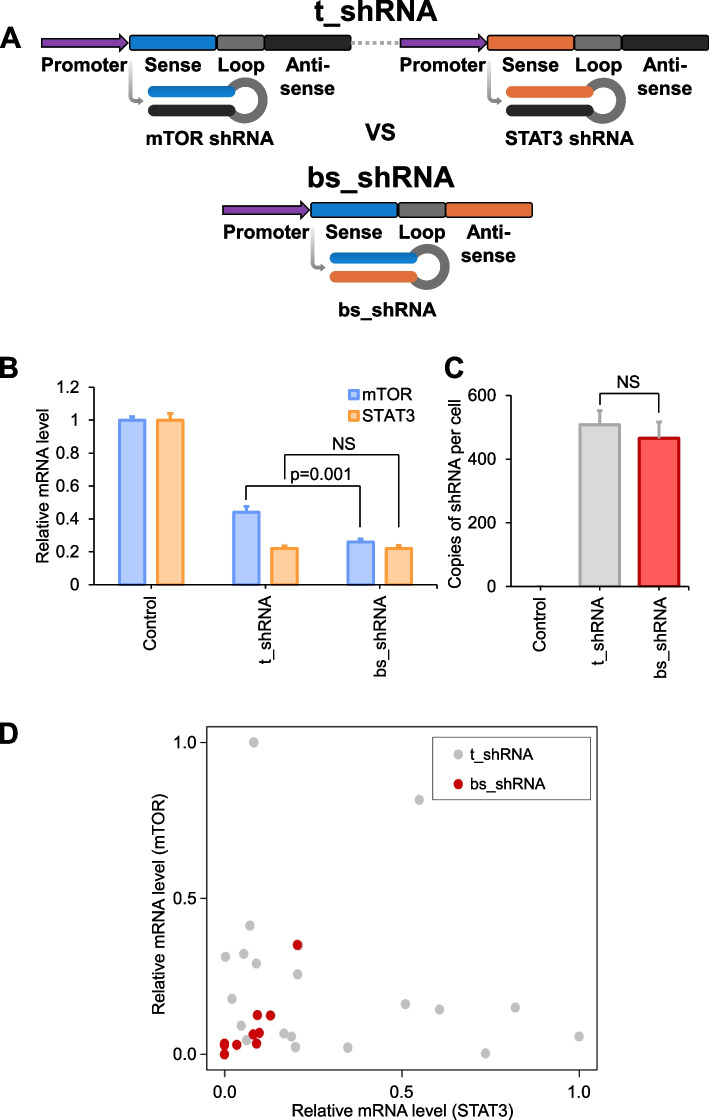
Balanced knockdown strategy for mTOR/STAT3 via an shRNA-mediated expression system. **A** Graphic illustration of the structure of the bispecific shRNA (bs_shRNA) compared with that of the conventional tandem shRNA system (t_shRNA). This system allows the encoding of two target sequences in a short coding length and decreases the off-target effect. **B** qPCR detection of mTOR and STAT3 expression of 253 J-BV for knock-down efficacy of t_shRNA and bs_shRNA in 253 J-BV transfected with t_shRNA and bs_shRNA. **C** The number of shRNA production of t_shRNA and bs_shRNA in 253 J-BV at a single cell level. Relative quantification is used through qPCR to calculate the copy number of the shRNA product. Refer to copy number calculation of shRNA in methods for detail method. **D** qPCR analysis at the single-cell level to compare the efficacy of knock-down between t_shRNA and bs_shRNA in each 253 J-BV transfected plasmid vector. Refer to RNA preparation and qPCR from a single cell in methods for detail method. (For statistics, two-tailed *t*-test for **B** and **C**, NS = non-significant)

Considering the implication of viral vectors, we intended to encode our siRNA as shRNA forms into the DNA plasmid to expect its natural formation after transcription. However, a further limitation of the conventional dual-targeting shRNA method is that cannot be used for multiple gene targeting. Because most traditional dual shRNAs are tandemly situated beneath distinct promoter sequences (the upper part of the graphic in Fig. 3A, t_shRNA), the downregulating effect of shRNAs on each gene differs at the single-cell level, preventing the synergistic effect. However, our newly designed bispecific shRNA (bs_shRNA) can simultaneously function within a single cell (the lower part of the graphic in Fig. 3A), producing the desired synergistic effect under the control of the same promoter. The superiority of this system over the traditional dual-targeting approach can be studied by measuring the knockdown efficacy of mTOR and STAT3 of bs_shRNA relative to the tandemly located shRNA (t_shRNA) system (Fig. 3B). The efficacy of the bi-specifically targeting shRNA (bs_shRNA) surpassed that of the previous system. We further demonstrated the mechanistic efficiency of the bs_shRNA system by quantifying shRNA productivity. The total shRNA productivity of the dual-targeting shRNA at the single-cell level was similar to that of the traditional tandemly-coded method depicted in the left panel of Fig. 3B (Fig. 3C). Nevertheless, at the single-cell level, t_shRNA typically uses a dual-promoter system to express different shRNA sequences. This structural discrepancy demonstrated although t_shRNA could not target both genes simultaneously within the scope of a single cell, bs_shRNA downregulated both target genes simultaneously at a well-balanced level (Fig. 3D).

### Incorporation of Bispecific shRNA into replication-competent adenovirus

**Fig. 4:**
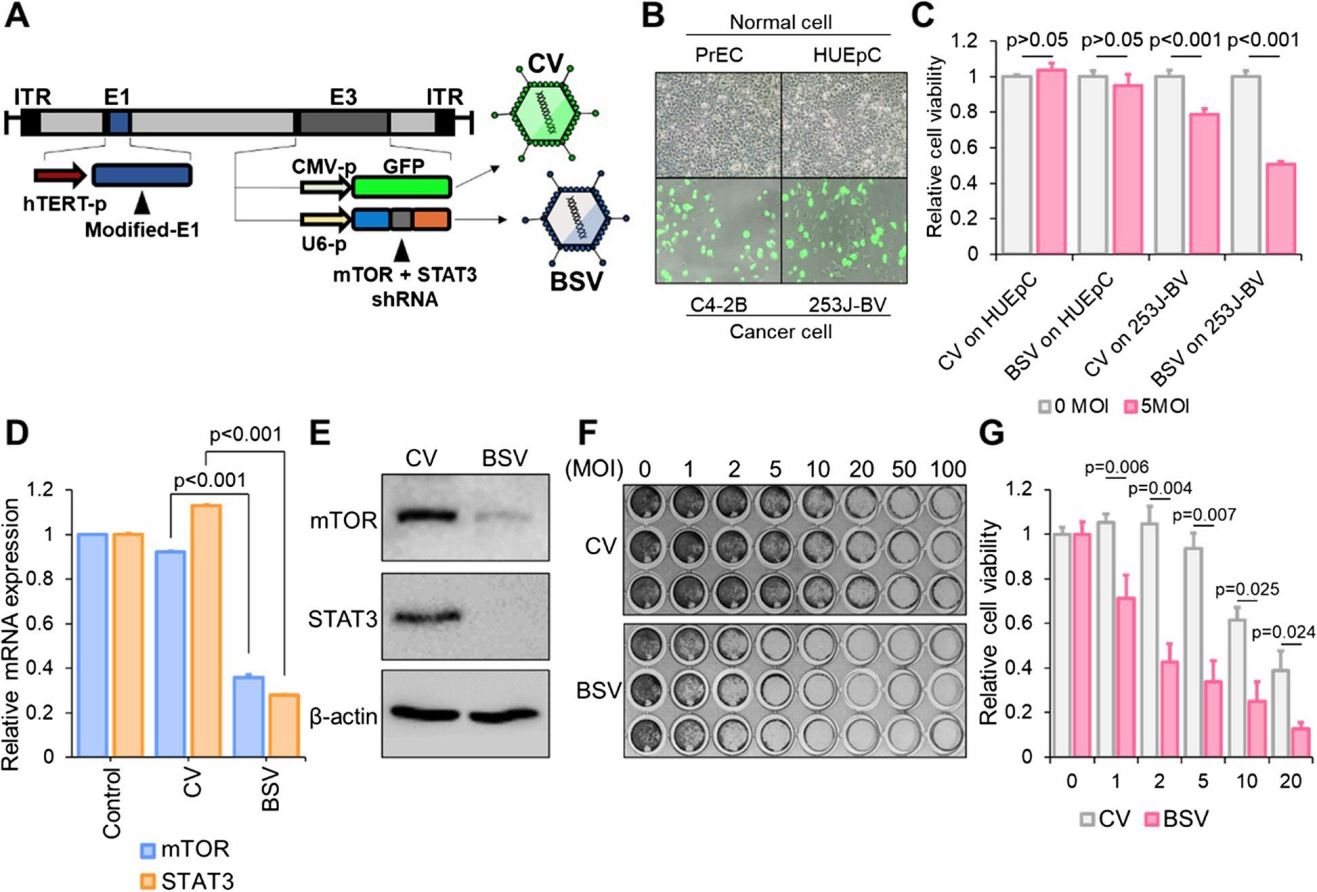
Incorporation of bispecific shRNA into replication-competent adenovirus. **A** Genetic construction of bispecific shRNA (bs_shRNA)-expressing adenovirus (BSV). The human telomerase promoter was encoded in the front of E1A-IRES-E1B, and the U6 promoter was used for the shRNA expression in E3. In the CV construct, the shRNA cassette under the U6 promoter was replaced with a GFP cassette driven by the CMV promoter. Refer to preparation of replication-competent adenovirus in methods for detail method. **B** Normal cells (PrEC and HUEpC) and cancer cells (C4-2B and 253 J-BV) were infected by 20 MOI of CV for 72 h. **C** Viral vector concentration (MOI)-based cell viability test: HUEpC and 253 J-BV cells were treated with 5 MOI of CV and BSV for 72 h. **D** Suppression of the expression of mTOR and STAT3 as indicated by real-time PCR. For this analysis, 253 J-BV cells were treated with 5 MOI CV and BSV for 72 h. **E** Western blotting revealing the changes between BSV- and CV-induced mTOR and STAT3 downregulation following the treatment of 253 J-BV cells with 5 MOI CV and BSV for 72 h. **F**, **G** Viral vector concentration (MOI)-based cell viability test using crystal violet staining (**F**) and cell viability assay (**G**). The 253 J-BV cells were treated with viruses for 72 h in a concentration-dependent manner (for statistics, two-tailed *t*-test for **C**, **D**)

To surmount the low delivery efficiency of RNAi therapy in vivo, we integrated the bispecific shRNA into an adenoviral vector which is specifically designed to replicate in cancer cells using the hTERT promoter. This bs_shRNA expressing sequence construct consists of two guide sequences and hairpin structures, which are inserted downstream of the U6 promoter. As a comparative control (adenovirus control, CV), sequences encoding green fluorescent protein (GFP) were inserted in place of the bispecific shRNA sequences (Fig. 4A).

We evaluated the safety of this viral vector system by assessing the cancer cell-specific infection of a basic oncolytic adenovirus control (CV), having an identical capsid without shRNA expression and a bispecific shRNA-expressing adenovirus (BSV) (Fig. 4B, C). Neither CV nor BSV exhibited any cytotoxic effect on human prostate epithelial cells (PrECs) and human urethra epithelial cells (HUEpCs) used as a control. However, both CV and BSV demonstrated selective cytotoxicity toward C4-2B, a prostate cancer cell line, and 253 J-BV, a bladder cancer cell line. Afterward, we ascertained that the BSV effectively downregulated both mTOR and STAT3 compared to CV using real-time PCR (Fig. 4D) and western blotting (Fig. 4E). We subsequently stained and quantified CV- and BSV-treated cells using crystal violet dye to validate BSV’s efficacy (Fig. 4F).

### Transcriptome analysis to ascertain the impact of BSV on cellular signaling pathways

**Fig. 5:**
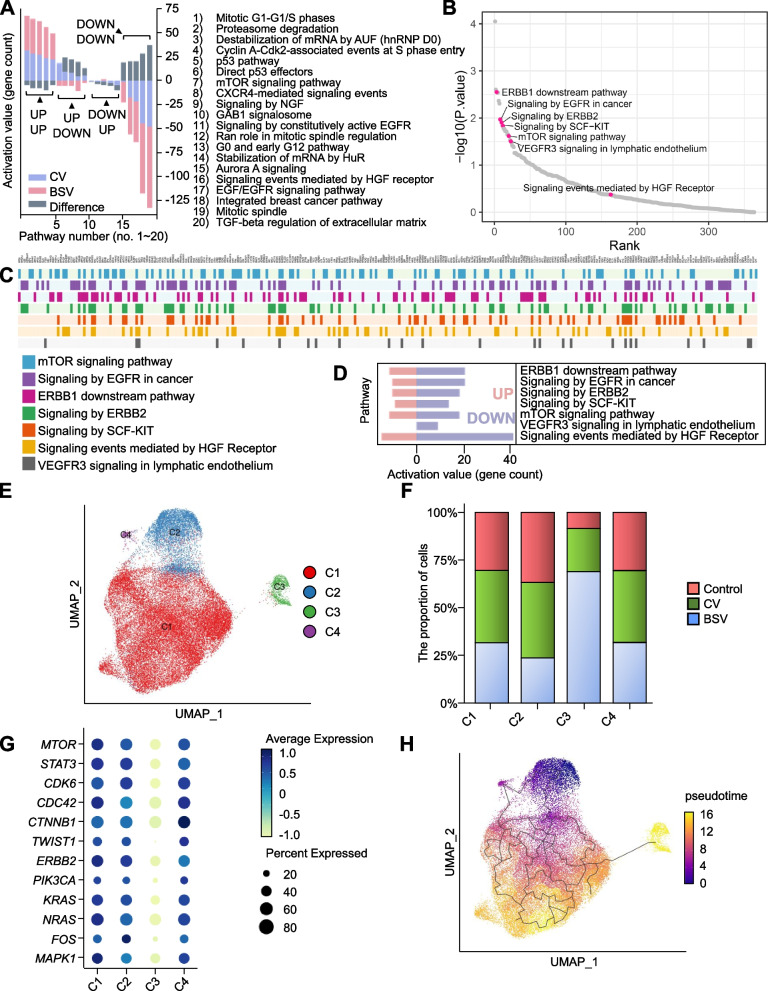
Transcriptome analysis to ascertain the impact of BSV on cellular signaling pathways. **A-D** An oncolytic adenovirus-armed bs_shRNA system was constructed and its potency was confirmed using RNA seq. Cells were analyzed after 72 h of 5 MOI virus infection. **A** Analysis of RNA sequencing-based signaling pathways affected by CV and BSV in A549 cells. **B** RNA sequencing results of (**A**), BSV-affected pathways were aligned using a volcano plot based on the log squared *p*-value. The pink dot refers to the representative significant-signaling pathway in cancer prognosis. **C** The seven most significant cancer pathways were selected and imaged to display every gene in each pathway. **D** The number of up- and down-regulated genes was counted in the selected pathways (**C**). **E** 253 J-BV cells were harvested after 72 h of 5 MOI virus infection (CV and BSV). Next, scRNA-seq was performed using the BD rhapsody platform. Single cells are displayed in the UMAP plot colored by clusters. **F** Bar plots showing the proportions of clusters according to the clusters. **G** Dot plot showing the average expression of representative markers for each cluster. **H** Single-cell trajectory colored by pseudotime. Refer to RNA sequencing and data analysis, and single-cell RNA seq and data processing in methods for detail method

BSV induced cell death in bladder cancer cells at remarkably low MOI (MOIs 1 and 2). We conducted RNA sequencing in A549 lung cancer cells to verify BSV’s downstream pathway (Fig. 5A). Both cell death and proliferation-related pathways were highly affected by BSV treatment, suggesting these to be consequences of mTOR and STAT3 knockdown. The epidermal growth factor receptor (EGFR) family ErbB1 and ErbB2, stem cell factor (SCF)-KIT, and vascular endothelial growth factor receptor 3 (VEGFR3) pathways, major pathways related to cancer aggressiveness (Fig. 5B), were downregulated. Particularly, the hepatocyte growth factor (HGF) receptor and vascular endothelial growth factor (VEGF) receptor pathways were found to be high-ranked pathways (Fig. 5C, D). In conclusion, BSV deactivated cancer-related pathways in a multi-directional manner (Fig. 5D).

One of the challenges while infecting the cells by a virus in vitro, is maintaining an even infection rate per cell. Moreover, dead cells in the sample impede the analysis of gene expression. Consequently, we focused on cells in a pre-death state to analyze the maximum effect of BSV bladder cancer. After dead cell elimination, single-cell RNA sequencing (scRNA-seq) was performed to sort out live cells 72 h after infection with CV or BSV, which were analyzed further.

The average silhouette width with the k-means clustering algorithm (Additional file 1: Fig. S1A) was used to divide single cells into four clusters (Fig. 5E), and their relative proportions were altered following treatment (Fig. 5F and Additional file 1: Fig. S1B). Compared to others, the C3 cluster exhibited a higher proportion of cells in the BSV group, indicating that C3 is the primary cluster affected by BSV infection. In line with the RNA-seq result of Fig. 5A–D, the expression of mTOR, STAT3, and several cancer-related markers was substantially downregulated in the C3 cluster (Fig. 5G). Pseudotime trajectory analysis identified a single-cell trajectory in the order of C2-C4-C1-C3, signifying a transcriptome transition according to the therapy (Fig. 5H). The C3 cluster, presumed to be infected cells by the therapy, was divided into two sub-clusters. High portion of control sample was belonged to S2 cluster, whereas High portion of BSV sample was belonged to S1 cluster (Additional file 1: Fig. S2A, B, C). The single-cell trajectory advanced from S2 to S1 along pseudotime, depending on the BSV infection (Additional file 1: Fig. S2D). The expression of several genes (Fig. 5G) supported the therapeutic effects, which were notably downregulated in the BSV sample (Additional file 1: Fig. S2E and F) and the S1 sub-cluster (Additional file 1: Fig. S2G). Remarkably, the CV sample exhibited a slightly reduced expression of these genes, which could be attributed to the infection by the oncolytic virus.

### Evaluation of in vivo effectiveness of BSV within a xenograft mouse model

**Fig. 6:**
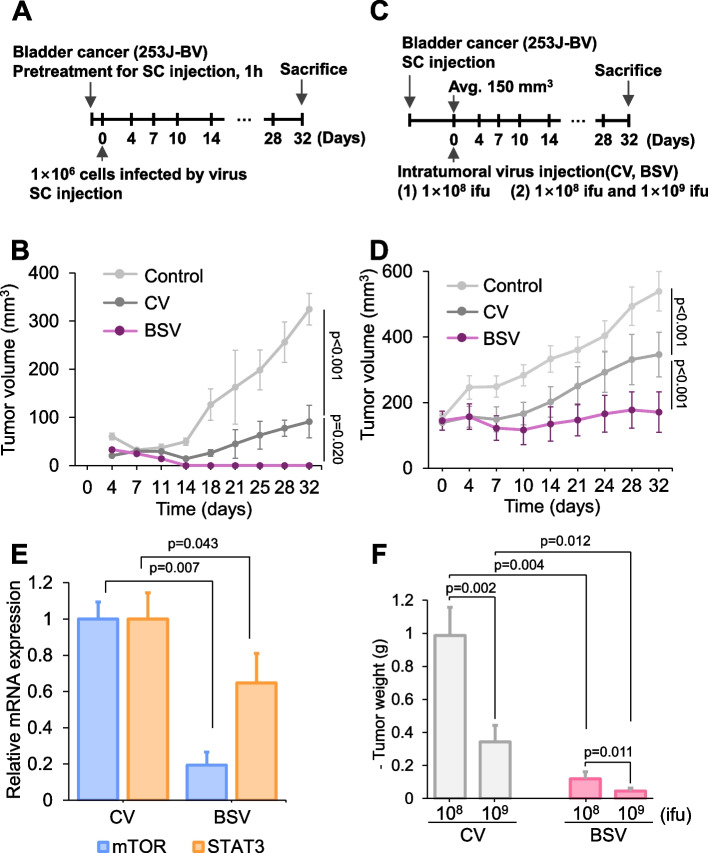
Evaluation of in vivo effectiveness of BSV within a xenograft mouse model. **A** Experimental schedule evaluating the efficacy of BSV ex vivo. **B** Tumor growth was recorded. 253 J-BV cells were infected at 2 MOI and subsequently injected subcutaneously in BALB/c nude mouse (1 × 10^6^ cells). Next, 1 × 10^6^ 253 J-BV cells were infected at 2 MOI of each virus and subsequently injected subcutaneously in BALB/c nude mouse (*n* = 6 mice for each group) at day 0. From day 4, tumor size was measured twice a week until day 32. **C** Experimental schedule evaluating the efficacy of BSV in vivo. Briefly, 1 × 10^6^ 253 J-BV cells were injected subcutaneously in mice. Viruses were injected intratumorally with 150 mm^3^ volume following the number of virus on the number 1 (as (1) 1 × 10^8^ IFUs, for (D)) and the number 2 (as (2) 1 × 10^8^ IFUs and 1 × 10^9^ IFUs, for (**F**)). **D** Tumor growth was recorded. A total of 1 × 10^6^ 253 J-BV cells were injected subcutaneously in mice (*n* = 6 mice for each group). Viruses (1 × 10^8^ IFUs) were injected intratumorally with 150 mm^3^ volume at day 0. Tumor size was measured twice a week for 32 days. **E** The mRNA levels of mTOR and STAT3 were measured using qPCR in the isolated tumors of (B) at day 32. **F** Based on (C), viruses (1 × 10^8^ and 1 × 10^9^ IFUs) were injected intratumorally and the tumors were established with 253 J-BV (1 × 10.^6^ cells). At day 32, tumor volume was measured after animal sacrifice (*n* = 6 mice for each group) (for statistics, two-tailed *t*-test for **B**, **D**, **E**, and **F**)

We ascertained the efficacy of the shRNA system in an animal model, for which we initially conducted ex vivo experiments using 253 J-BV cells. Cells were treated with the virus before harvesting to achieve 100% infection. Subsequently, the cells were collected and inoculated into animals (Fig. 6A). We monitored the tumor size every 3 or 4 days. We observed that although CV exhibited a 50% tumor-killing effect, tumors did not form in animals inoculated with BSV-treated cells (Fig. 6B). Next, we designed an experiment more similar to clinical situations. We transplanted bladder cancer cells into an immunodeficient mouse model and waited for the tumor to form and grow to a certain size. Afterward, CV or BSV was intratumorally injected (Fig. 6C). We found that a single injection significantly constrained the tumor growth compared to CV- or buffer-treated groups (Fig. 6D). In addition, we measured the mRNA levels using samples from the same batch and found that BSV successfully reduced the mRNA levels of mTOR and STAT3 in vivo (Fig. 6E). Also, we measured the tumor volume on the last day of the experiment under different concentrations of the viral vector (described in Fig. 6C as (2)) and found that it reduced in a dose-dependent manner (Fig. 6F).

### Confirmation of potential as a novel therapeutic candidate for bladder *cancer* treatment

**Fig. 7:**
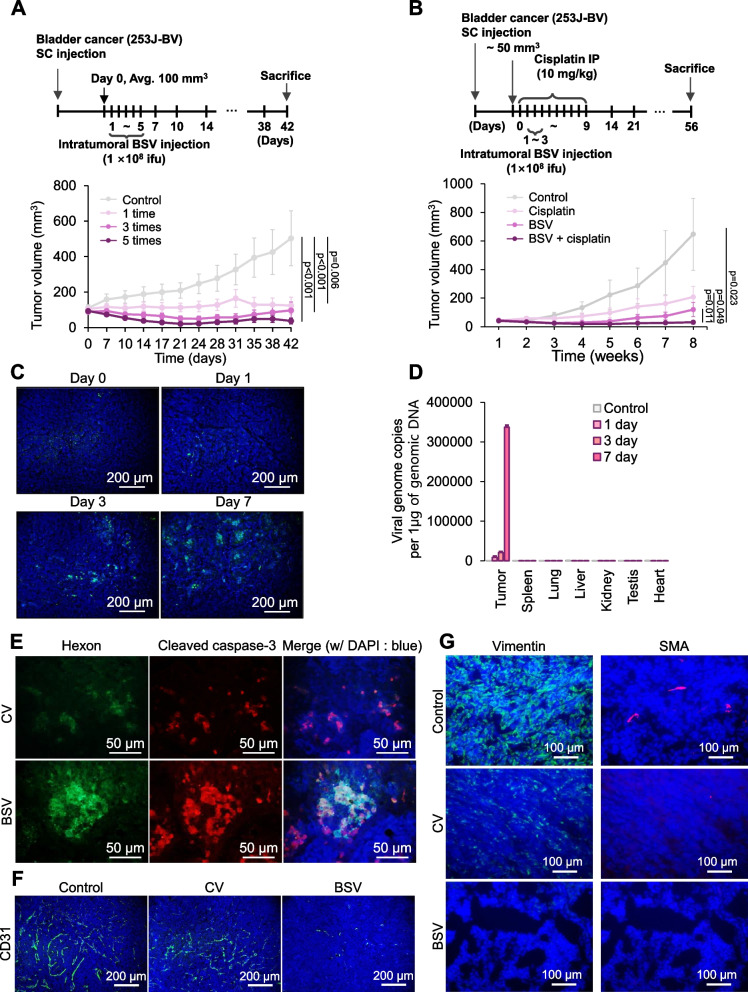
Confirmation of potential as a novel therapeutic candidate for bladder cancer treatment. **A** Schematic of the timeline for multiple virus treatments at the upper panel. A total of 1 × 10^6^ 253 J-BV cells were injected subcutaneously in mice (*n* = 6 mice for each group). When the tumor volume reached 100 mm^3^ (day 0), BSV (1 × 10^8^ IFUs) and CV (1 × 10^8^ IFUs) were intratumorally multi-injected (only BSV, 1, 3, or 5 times) in the initial 3 or 5 days (1 time: day 1; 3 times: days 1, 2 and 3; 5 times: days 1, 2, 3, 4, and 5). At the under panel, tumor volume was measured twice a week for 7 weeks. **B** A total of 1 × 10^6^ 253 J-BV cells were injected subcutaneously in mice (*n* = 6 mice for each group). When the tumor volume reached 50 mm^3^ (day 0), BSV (1 × 10^8^ IFUs) were intratumorally injected from days 1 to 3 daily. Mice were treated daily with a combination of cisplatin (10 mg/kg, intraperitoneal injection) from days 0 to 9. The tumor was measured once every week. Tumor volume curve at the under panel. **C-D** A total of 1 × 10^6^ 253 J-BV cells were subcutaneously injected into BALB/c nude mice. When the tumor reached a volume of 100 mm^3^, an intratumoral injection of 1 × 10^8^ IFUs of CV was administered (designated as day 0). On the first, third, and seventh days subsequent to the CV injection, both the tumors and other organs (including the spleen, lungs, liver, kidneys, testis, and heart) were harvested to assess viral distribution. **C** GFP (green) and nuclei (blue) were visualized using an anti-GFP antibody and DAPI, respectively, during immunofluorescence staining. The tumors were harvested on days 0, 1, 3, and 7. **D ***E1A* gene, known as a viral gene, was analyzed within the tumor and each organ (spleen, lungs, liver, kidneys, testis, and heart) to evaluate the distribution of distribution. Refer to biodistribution analysis for intravesical BSV instillation in methods for detail method. **E–G** A total of 1 × 10^6^ 253 J-BV cells were injected subcutaneously in BALB/c nude mice. When the tumor volume reached 100 mm^3^, 1 × 10.^8^ IFUs of CV and BSVwere injected intratumorally. After 7 days for (E) and 14 days for (**F** and **G**), mice were euthanized and tumors were harvested for analysis. **E** Hexon (green) and cleaved caspase-3 (red) were stained by immunofluorescence staining for these expressions. **F** Vasculature was visualized using an anti-CD31 antibody for immunofluorescence staining.** G** Vimentin (green) and smooth muscle actin (red), known as epithelial–mesenchymal transition (EMT) markers, were stained to evaluate the inhibitory effect of metastasis of BSV (for statistics, two-tailed *t-*test for **A** and **B**)

We performed repeated injections and co-treatment with cisplatin, as illustrated in Fig. 7A (upper panel) and 7B (upper panel), respectively. We noted that repeated multiple injections during the initial 5-day period significantly reduced the tumor volume (Fig. 7A, at the lower panel). We discovered that cisplatin displayed a moderate therapeutic effect, whereas co-treatment with BSV completely eliminated the tumor at 7 weeks post-treatment (Fig. 7B at the lower panel).

We next assessed the cancer cell-specific infection of this viral system and its safety by observing viral spread within the tumor using the oncolytic adenovirus control (CV) at different time points (days 0, 1, 3, and 7) (Fig. 7C). In addition, we analyzed the distribution of CV within different organs (Fig. 7D). The results obtained from these experiments conclusively demonstrated that this oncolytic viral carrier exclusively infected cancer cells while sparing normal cells. Histologically, hexon-stained regions overlapped with cell death signals marked by cleaved caspase-3 (Fig. 7E). Considering the area of hexon staining and the intensity of the cleaved caspase-3 signal, BSV caused more potent viral propagation and subsequent cell death compared to CV (Fig. 7E). Furthermore, cells infected with BSV exhibited reduced vascular endothelial growth factor receptor (VEGFR) signaling compared to those infected with CV, as indicated by the RNA sequencing data (Fig. 5A-D). As anti-angiogenesis is one of the markers used to determine the efficacy of cancer therapy, we assessed vessel development in the tumor region using CD31 immunostaining (Fig. 7F). As anticipated, vasculature was well detected in the control; however, it was weak in CV-treated tumors. We found that BSV decreased the CD31-positive area by more than 90% compared to that in CV-treated tumors (Fig. 7F). Furthermore, we evaluated the levels of vimentin and smooth muscle actin (SMA) to determine metastatic aggressiveness. Notably, BSV-treated tumors displayed decreased levels of both vimentin and SMA (Fig. 7G).

### Evaluation of in vivo effectiveness of BSV within an orthotopic bladder *cancer* mouse model

**Fig. 8:**
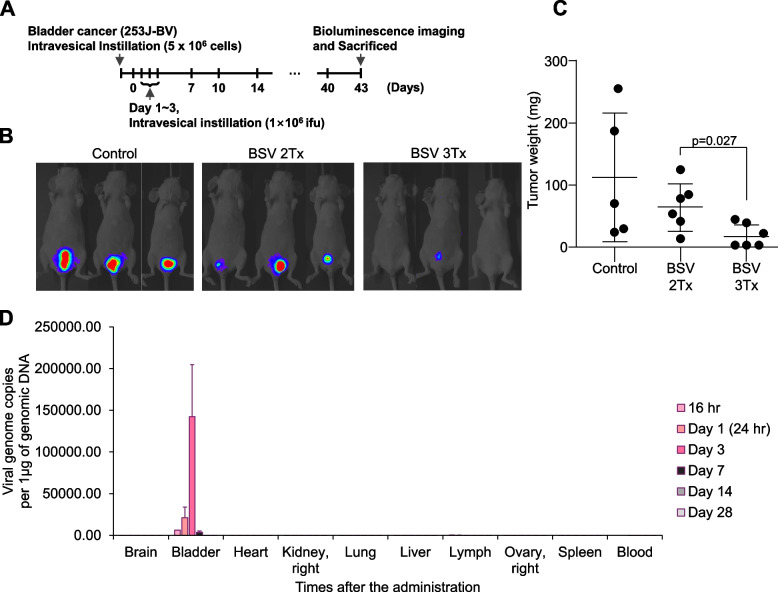
Evaluation of the in vivo effectiveness of BSV within an orthotopic bladder cancer mouse model. **A** A schedule scheme for intravesical instillation based orthotopic animal experiment. Seven days after the instillation of 5 × 10^6^ 253 J-BV-Luc cells, viruses (1 × 10^8^ IFUs) were injected on days 1, 2, and 3. Viruses were injected into the mice in BSV 2Tx group at days 1 and 2, and in BSV 3Tx group at days 1, 2, and 3. At day 43, tumor growth was visualized using bioluminescence imaging, and tumor samples were harvested after sacrifice (*n* = 5 mice for control, *n* = 6 mice for BSV 2Tx and BSV 3Tx). **B** Tumor size of (**A**) was visualized by bioluminescence imaging. **C** Isolated tumors (**A**) were weighed (for statistics, two-tailed *t*-test for **C**). **D** The biodistribution analysis for intravesical BSV instillation within internal organs was investigated over time using a hamster model that received intravesical instillation of BSV

We established an orthotopic animal model by injecting bladder cancer cells (253 J-BV-Luc) using intravesical instillation. After 7 days of intravesical instillation of 253 J-BV-Luc, BSV was administered into the bladder cavity by intravesical instillation daily from day 1 to day 3. At day 43, tumors were visualized using bioluminescence imaging (Fig. 8A). Luminescence intensity was relatively weak in the BSV-treated groups (Fig. 8B), and tumor weight significantly decreased following BSV administration (Fig. 8C). The spatiotemporal biodistribution of BSV was examined using an intravesical instillation model to assess the unintended adverse effects of BSV through off-target organ distribution (Fig. 8D).

### BSV facilitates CD8^+^ T-cell recruitment into the tumor in a humanized mouse model

**Fig. 9:**
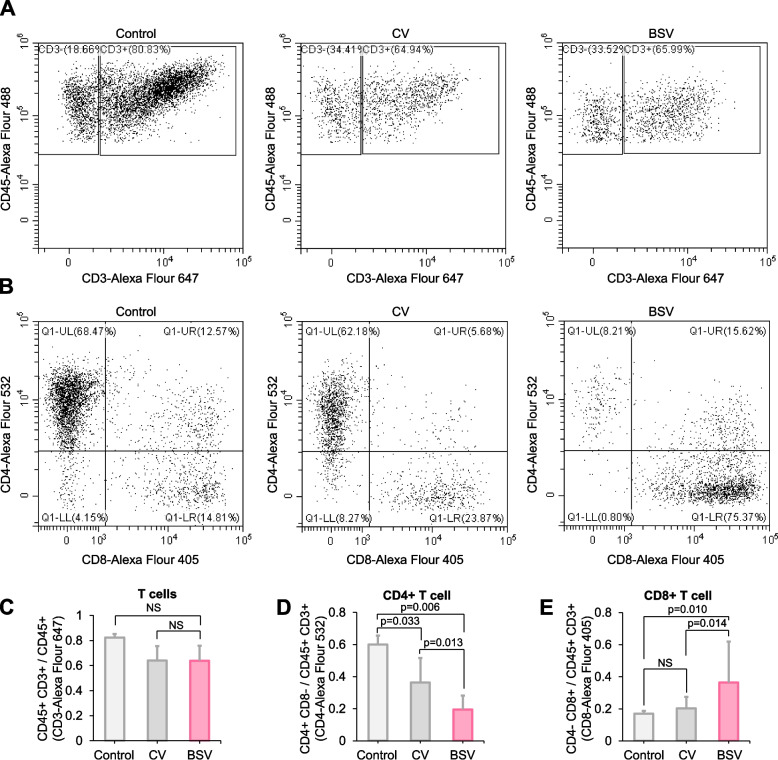
BSV facilitates CD8^+^ T-cell recruitment into the tumor in a humanized mouse model. **A-E** The population of immune cells was analyzed following a viral treatment; CD34 + hu-NSG (*n* = 6, each group) humanized animals were used. 253 J-BV cells were subcutaneously inoculated. The virus was intratumorally injected at the 60 mm^3^ tumor volume. The population of immune cells was monitored at 21 days after viral injection. **A** Representative plot of flow cytometry for determining T-cell population. **B** Representative plot of flow cytometry for determining CD4^+^ or CD8^+^ T-cell population. **C** T-cell populations were compared using CD45^+^/CD3^+^ by flow cytometry based on (**A**). **D** CD4^+^ T-cell populations were compared using CD45^+^/CD3^+^/CD4^+^/CD8^−^ by flow cytometry based on (**B**). **E** CD8^+^ T-cell populations were compared using CD45^+^/CD3^+^/CD4^−^/CD8.^+^ by flow cytometry based on (**B**) (for statistics, two-tailed *t*-test for **C**, **D**, **F**-test for **E**, NS = non-significant)

We attempted to identify any BSV-induced changes in the immune-related microenvironment. We dissociated tumors and analyzed infiltrated immune cells in a 253 J-BV xenograft model of humanized mice using flow cytometry (Fig. 9A, B). Although the number of T-cell populations did not significantly change among the treatment groups (Fig. 9A, C), BSV decreased the population of CD4-positive cells (Fig. 9B, D), whereas the number of CD8-positive cells increased in the tumor compared to CV-treated tumors (Fig. 9E). We obtained comparable results in the PBMC-transplanted, tumor-bearing nude mouse model (Additional file 1: Fig. S5A-D). Also, an increase in M1 macrophage infiltration and a decrease in M2 macrophage infiltration were observed in the BSV-treated group compared to the CV group (Additional file 1: Fig. S5E). The M1 macrophage population was higher in the BSV-treated group, while the M2 macrophage population, which is associated with tumor-promoting activities, was lower. Consequently, the expression levels of TNFα and IL-6, cytokines released by M1 macrophages, were elevated in the BSV-treated group (Additional file 1: Fig. S5F).

## Discussion

Although the pursuit of multi-gene targeting presents a significant advancement in tumor medications, the scope of selectable targets remains confined predominantly only to “druggable” targets. Several potential targets of substantial efficacy are yet to be identified from the undruggable realm. siRNAs have been considered a relatively new modality to cure cancer by downregulating cancer-driving genes. We designed a bispecific shRNA system that successfully matched target sequences of two different poor prognosis-associated genes, namely, mTOR and STAT3, to improve the low efficacy of single-target RNAi.

Especially, mTOR is considered a suitable target for RNAi applications. mTOR exists in two distinct complexes, mTORC1 and mTORC2. mTOR-targeting siRNA effectively suppresses both mTOR complexes, unlike current chemical-based mTOR inhibitors such as rapamycin, which selectively target only mTORC1. This selective inhibition leads to aberrant activation of mTORC2 as a compensatory response [27, 28]. Consequently, activation of mTORC2 results in phosphorylation of Akt at the S473 site, ultimately promoting cell survival. In light of this, we observed inhibition of Akt phosphorylation at the S473 site via RNAi treatment (Fig. 2E). Furthermore, RNA sequencing data indicated that other major downstream pathways of mTORC2, including VEGF, EGFR, ERBB, and SCF-KIT, are potentially affected by mTOR inhibition [29, 30](Fig. 5 and Additional file 1: Fig. S2F, G). These pathways are closely related to receptor-MAPK signaling (RAS-RAF-MEK-ERK), which also serves as a compensatory pathway downstream of mTOR inhibition [31].

We addressed the difficulty of intracellular delivery of a large nucleotide load required for RNAi therapy. We coded the target sequences into a viral vector, which was modified with DSG2 binding fiber and hTERT promoter [32], had a fail-safe system of tumor selectivity to avoid the toxicity of general gene therapy, and could be produced as a short hairpin structure. The replication of the viral vector was associated with an increased number of shRNAs (Fig. 3C). Interestingly, complete repression of tumor growth was observed in 100% infected conditions by ex vivo treatment. Therefore, an increase in the infection rate in vivo could consequently enhance the efficacy. The infection yield in vivo could be optimized by several modifications of the viral vector. Further, ex vivo and in vivo treatments of the oncolytic virus lacking the encoded dual shRNA sequence demonstrated limited therapeutic effect (Fig. 6A-D). These results indicated clinical limitations of a single-dose treatment of current oncolytic virus therapies lacking therapeutic loads with both divergent and complementary mechanisms of action.

Moreover, BSV coding sh-mTOR and STAT3 activated CD8^+^ T cells differently from the non-shRNA coding virus treatment, suggesting that the diverging CD8^+^ T cell pattern was induced by the downregulation of STAT3, mTOR, or possibly both targets. The inhibition of VEGF boosted the efficacy of immune checkpoint inhibitors [33], whereas GM-CSF is a key regulator responsible for T-cell activation. STAT3 upregulates cancer cell-derived VEGF [34], and expression of GM-CSF is partially responsible for mTOR signaling [35]. Thus, bispecific targeting of mTOR and STAT3 could support increased BSV-induced infiltration of CD8^+^T cells into the tumor region more significantly than the oncolytic virus treatment itself. These findings suggest that the modified Ad5/3 system used could be a strong future candidate in combination with immune checkpoint inhibitors (ICIs).

mTOR and STAT3 were selected as the two gene targets for a knockdown in our pilot system based on bladder cancer patient data (Figs. 1 and 2). Specifically, patients with bladder cancer having lower levels of mTOR and STAT3 were predicted to have a longer survival compared to those with high mTOR and STAT3 levels. Although it is not invariably the case that a gene, whose overexpression is correlated with diminished survival rates, will necessarily be an efficacious therapeutic target, several compelling targets have been identified and have been proven successful in numerous studies [36, 37]. Furthermore, the overexpression of the selected gene during a targeted therapy has often been used as a biomarker to identify appropriate patient cohorts for intervention [38]. This phenomenon could be ascribed to improved drug response enabled by restrained drug resistance [39, 40] or compromised metastatic properties such as invasiveness and inflammation that knockdown of the two molecules synergistically generate [41]. Thus, mTOR and STAT3 were determined to be not only effective survival markers but also promising therapeutic targets for simultaneous knockdown.

In conclusion, a unique therapeutic method at the interface between RNAi and oncolytic therapy was introduced. We propose that the two modalities could mutually complement and compensate for the respective shortcomings of each technique such as delivery of RNAi therapy and low in vivo efficacy of oncolytic viral treatment.

## Conclusion

The present investigation substantiates the efficacy of an oncolytic adenovirus-armed dual-targeting RNA interference (RNAi) approach for bladder cancer treatment. The nexus between mTOR and STAT3, and their association with adverse outcomes in patients with bladder cancer exhibiting elevated expression of both, is corroborated through comprehensive molecular analyses. Subsequently, this sequence is incorporated into an adenoviral vector, with its therapeutic potential being evaluated in a relevant animal model. The outcomes of this study reveal that the application of BSV transcends tumoral lysis, extending to a notably enhanced T-cell recruitment, thereby augmenting the anti-cancer response.

## Supplementary Information

Supplementary Material 1.

## Data Availability

All data generated and/or analyzed during the current study are available from the corresponding authors on reasonable request. Raw data of scRNA-seq and RNA-seq have been deposited in the NCBI as follows: Fig. 5E, H and Figs. S1 and S2, PRJNA922229; Fig. 5A-D, PRJNA922310. All other data supporting the findings of this study are available from the corresponding author on reasonable request. Source data are provided with this paper.

## Abbreviations

siRNA: Small Interfering RNA
mTOR: Mechanistic Target of Rapamycin
STAT3: Signal Transducer and Activator of Transcription 3
RNAi: RNA Interference
mTORC1: Mechanistic Target of Rapamycin Complex 1
mTORC2: Mechanistic Target of Rapamycin Complex 2
shRNA: Short Hairpin RNA
BCG: Bacillus Calmette-Guerin
NMIBC: Non-Muscle Invasive Bladder Cancer
GM-CSF: Granulocyte–Macrophage Colony-stimulating Factor
NCBI: National Center for Biotechnology Information
TCGA: The Cancer Genome Atlas
BSV: Bispecific shRNA-expressing Virus
CV: Control Virus
hTERT: Human Telomerase Reverse Transcriptase
IFU: Infectious Units
UMAP: Uniform Manifold Approximation and Projection
DEG: Differentially Expressed Gene
MOI: Multiplicity of Infection
FPKM: Fragments per kb per million reads
4E-BP-1: Eukaryotic Translation Initiation Factor 4E-binding Protein 1
FAK: Focal Adhesion Kinase
S6K1: Ribosomal Protein S6 Kinase Beta-1
AKT: Protein Kinase B (also known as PKB)
t_shRNA: Tandemly Located shRNA
bs_shRNA: Bi-specifically Targeting shRNA
GLP: Good Laboratory Practice
HUEpC: Human Urethra Epithelial Cells
EGFR: Epidermal Growth Factor Receptor
VEGFR3: Vascular Endothelial Growth Factor Receptor 3
HGF: Hepatocyte Growth Factor
VEGF: Vascular Endothelial Growth Factor
scRNA-seq: Single-cell RNA Sequencing
VEGFR: Vascular Endothelial Growth Factor Receptor
SMA: Smooth Muscle Actin
DSG2: Desmoglein 2
