# Correction: Intravesical instillation-based mTOR-STAT3 dual targeting for bladder cancer treatment

**DOI:** 10.1186/s13046-025-03469-6

**Published:** 2025-07-17

**Authors:** Dae Hoon Lee, Jung Ki Yoo, Ki Hwan Um, Wootae Ha, Soo Min Lee, Junseong Park, Min Jeong Kye, Jungyo Suh, Jin Woo Choi

**Affiliations:** 1 Department of Pharmacology, College of Pharmacy, Kyung Hee University, Seoul, 02447 Republic of Korea; 2 Department of Biomedical and Pharmaceutical Sciences, College of Pharmacy, Kyung Hee University, Seoul, 02447 Republic of Korea; 3 R&D Center of Curigin Ltd, Curigin, Seoul, 04778 Republic of Korea; 4 Department of Regulatory Science, College of Pharmacy, Kyung Hee University, Seoul, 02447 Republic of Korea; 5 Precision Medicine Research Center, College of Medicine, The Catholic University of Korea, Seoul, 06591 Republic of Korea; 6 Department of Urology, Asan Medical Center, University of Ulsan College of Medicine, Seoul, 05505 Republic of Korea

**Correction** **:** **J Exp Clin Cancer Res 43, 170 (2024)**

**https://doi.org/10.1186/s13046-024-03088-7**

Following publication of the original article [1], the authors identified two errors in manuscript. One is about method section which the sequence information was incorrect, and the other is Fig. 4A which shows incorrect promoter information for GFP. The correct information is presented blow:

**Incorrect sequences:**

1. t_shRNA (RNA sequence: GUG GCA UCC A CC UGC AUU U/GAG GCG CCU ACC UGC AUU U and AUG CAG GUG GAU GCC ACU U/AUG CAG GUA GGC GCC UCU U)
2. bs_shRNA (RNA sequence: GUG GCA UCC ACC UGC AUU U/AUG CAG GUA GGC GCC UCU U)
3. bispecific shRNA sequence (GTGGCATCCACCTGCATTTGGATCCAAATGCAGGTAGGCGCCTCTT)

**Correct sequences:**

1. t_shRNA (RNA sequence: GAC UGU GGC AUC CAC CUG CAU = UU/GAC UGA GGC GCC UAC CUG CAU = UU and AUG CAG GUG GAU GCC ACA GUC = UU/AUG CAG GUA GGC GCC UCA GUC = UU)
2. bs_shRNA (RNA sequence: GAC UGU GGC AUC CAC CUG CAU = UU/AUG CAG GUA GGC GCC UCA GUC = UU)
3. bispecific shRNA sequence (GACTGTGGCATCCACCTGCATTTGGATCCAAATGCAGGTAGGCGCCTCAGTCTT)

**Incorrect Fig.** 4

**Fig. 4:**
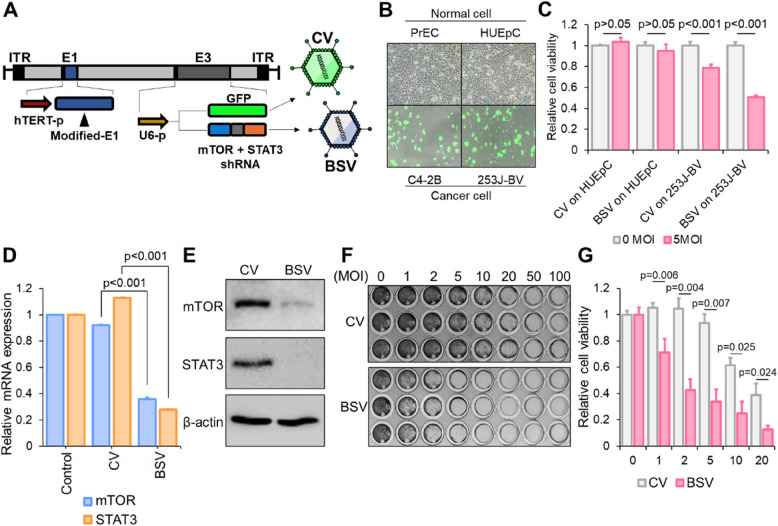
Incorporation of bispecific shRNA into replication-competent adenovirus **A** Genetic construction of bispecific shRNA (bs_shRNA)-expressing adenovirus (BSV). The human telomerase promoter was encoded in the front of E1A-IRES-E1B, and the U6 promoter was used for the shRNA expression in E3. In CV, the shRNA coding region was replaced by a GFP-coding sequence. Refer to preparation of replication-competent adenovirus in methods for detail method. **B** Normal cells (PrEC and HUEpC) and cancer cells (C4-2B and 253 J-BV) were infected by 20 MOI of CV for 72 h. **C** Viral vector concentration (MOI)-based cell viability test: HUEpC and 253 J-BV cells were treated with 5 MOI of CV and BSV for 72 h. **D** Suppression of the expression of mTOR and STAT3 as indicated by real-time PCR. For this analysis, 253 J-BV cells were treated with 5 MOI CV and BSV for 72 h. **E** Western blotting revealing the changes between BSV- and CV-induced mTOR and STAT3 downregulation following the treatment of 253 J-BV cells with 5 MOI CV and BSV for 72 h. **F**, **G** Viral vector concentration (MOI)-based cell viability test using crystal violet staining (**F**) and cell viability assay (**G**). The 253 J-BV cells were treated with viruses for 72 h in a concentration-dependent manner (for statistics, two-tailed *t*-test for **C**, **D**)

**Correct Fig.** 4

**Fig. 4:**
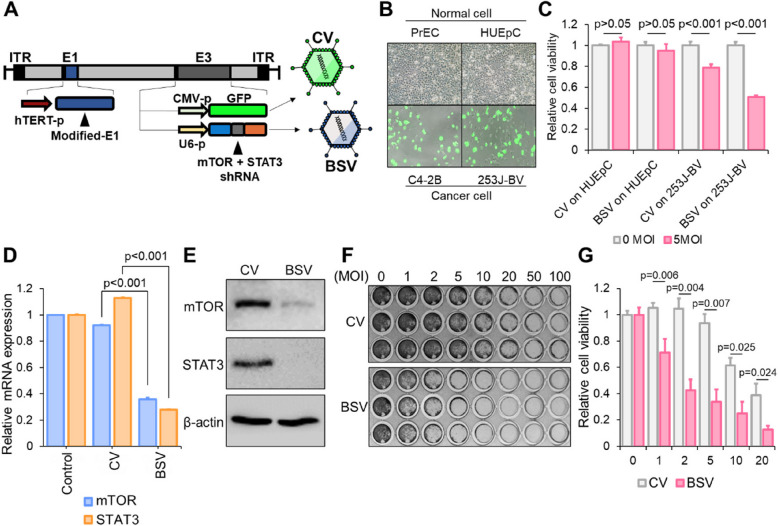
Incorporation of bispecific shRNA into replication-competent adenovirus **A** Genetic construction of bispecific shRNA (bs_shRNA)-expressing adenovirus (BSV). The human telomerase promoter was encoded in the front of E1A-IRES-E1B, and the U6 promoter was used for the shRNA expression in E3. In the CV construct, the shRNA cassette under the U6 promoter was replaced with a GFP cassette driven by the CMV promoter. Refer to preparation of replication-competent adenovirus in methods for detail method. **B** Normal cells (PrEC and HUEpC) and cancer cells (C4-2B and 253 J-BV) were infected by 20 MOI of CV for 72 h. **C** Viral vector concentration (MOI)-based cell viability test: HUEpC and 253 J-BV cells were treated with 5 MOI of CV and BSV for 72 h. **D** Suppression of the expression of mTOR and STAT3 as indicated by real-time PCR. For this analysis, 253 J-BV cells were treated with 5 MOI CV and BSV for 72 h. **E** Western blotting revealing the changes between BSV- and CV-induced mTOR and STAT3 downregulation following the treatment of 253 J-BV cells with 5 MOI CV and BSV for 72 h. **F**, **G** Viral vector concentration (MOI)-based cell viability test using crystal violet staining (**F**) and cell viability assay (**G**). The 253 J-BV cells were treated with viruses for 72 h in a concentration-dependent manner (for statistics, two-tailed *t*-test for **C**, **D**)

The correction does not compromise the validity of the conclusions and the overall content of the article. The original article [1] has been updated.
