# Supplementary material for: Intravesical instillation-based mTOR-STAT3 dual targeting for bladder cancer treatment
Source: J Exp Clin Cancer Res. 2024 Jun 18;43:170. doi: 10.1186/s13046-024-03088-7 (PMC11184849; doi:10.1186/s13046-024-03088-7)
Supplement: Supplementary file 1 — Supplementary Material 1. [file 13046_2024_3088_MOESM1_ESM.pdf]

Supplementary Data

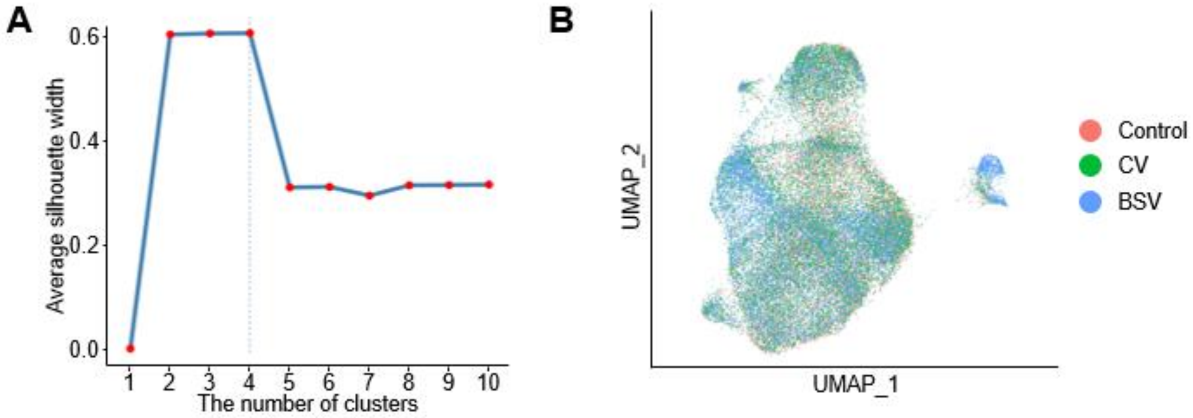

Supplementary Figure 1 Clustering of single-cell transcriptome.

A Average silhouette widths with k-means clustering (k = 1 to 10) of single-cell expression profiles.

B This figure is the part of figure 5E and F. UMAP plot colored by samples.

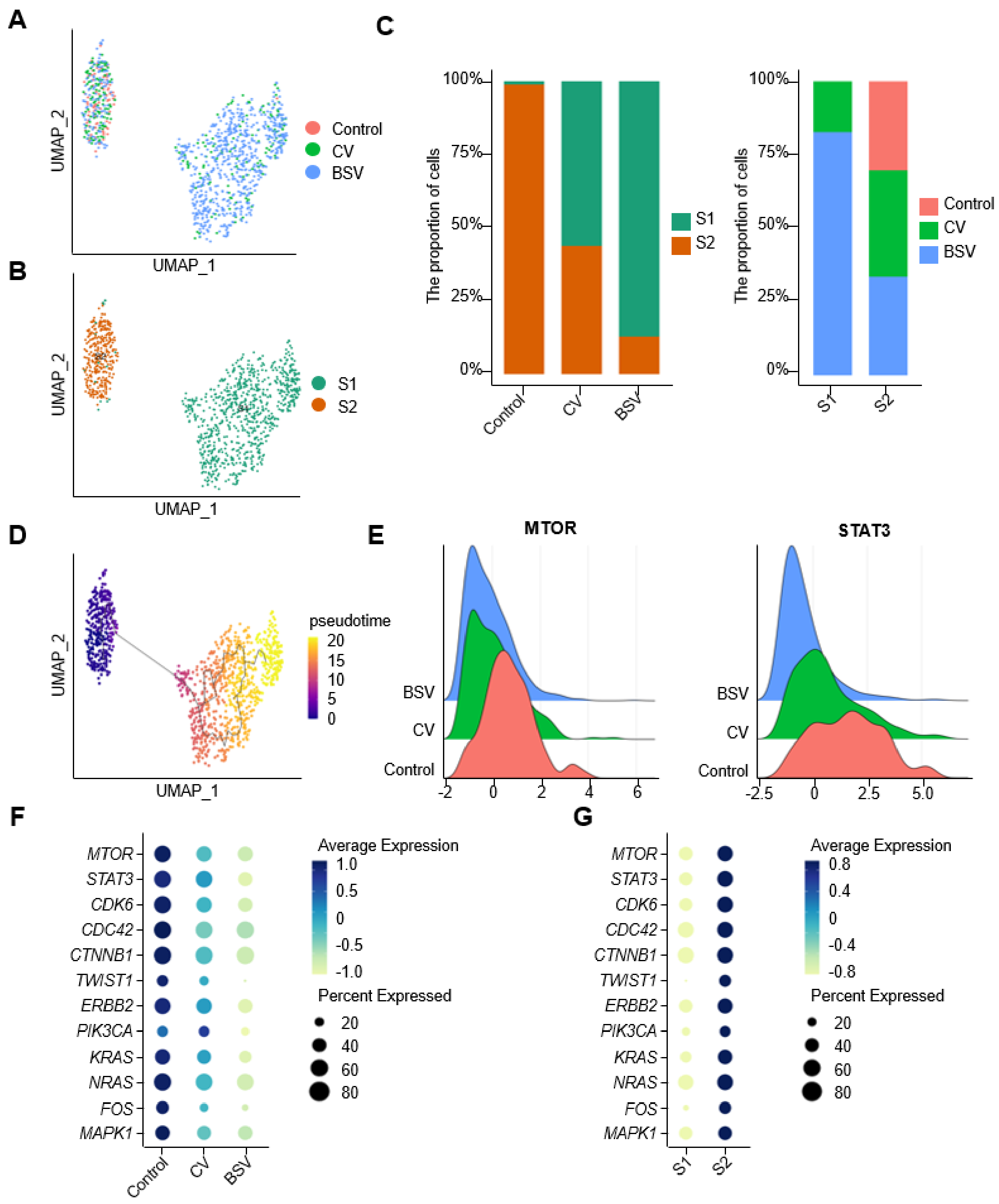

Supplementary Figure 2 Subset analysis of C3 cluster.

A-B UMAP plots of C3 cluster colored by groups (A) and sub-clusters (B).

C Bar plots showing the proportions of C3 sub-clusters according to the groups (left) and those of groups according to the sub-clusters (right).

D Single-cell trajectory of C3 cluster colored by pseudotime.

E Ridge plots showing mTOR and STAT3 expression in C3 cluster.

F-G Dot plot showing the average expression of representative markers for each sample (F) and sub-cluster (G).

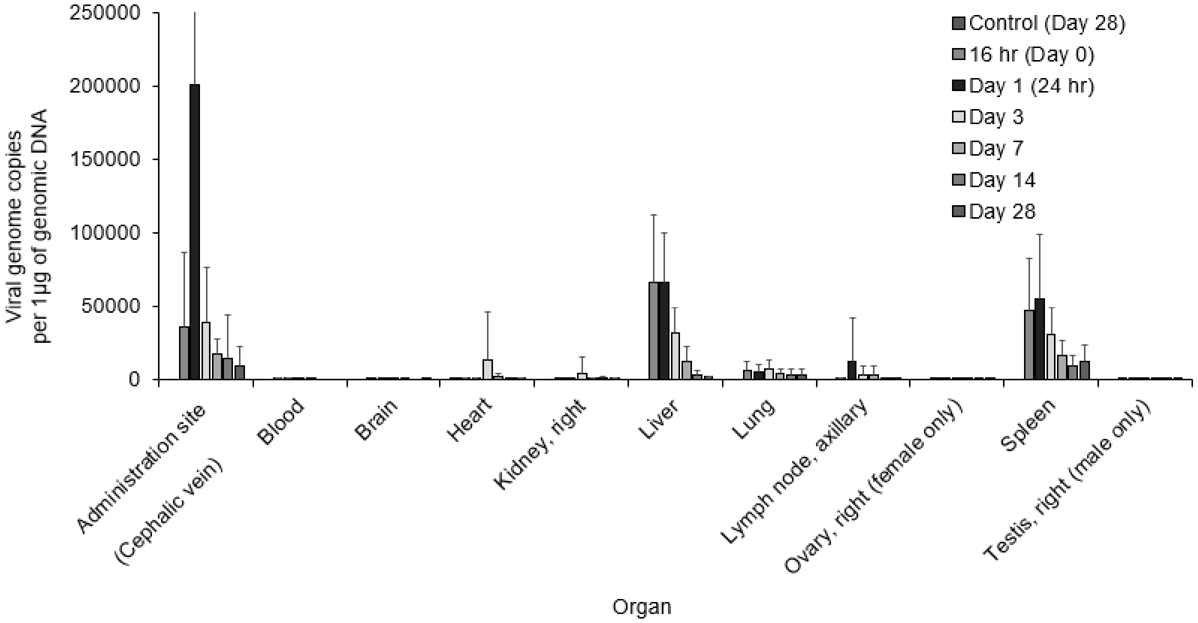

Supplementary Figure 3 The spatiotemporal distribution of BSV within internal organs was investigated over time by employing a hamster model that received intravenous single-shot injection model of BSV.

A total of $2.4x10^{10}$ ifu/kg of BSV were administered via intravenous injection in the hamster model. At each designated time point after administration, the animals within each group were humanely sacrificed, and their organs were subsequently analyzed for viral distribution. The genomic DNA from each organ sample was then extracted, and the number of viral particles was quantified based on the copies of viral genome. This study was conducted utilizing a sample size of n=5 hamsters per group, each of which was aged 8 weeks old.

A

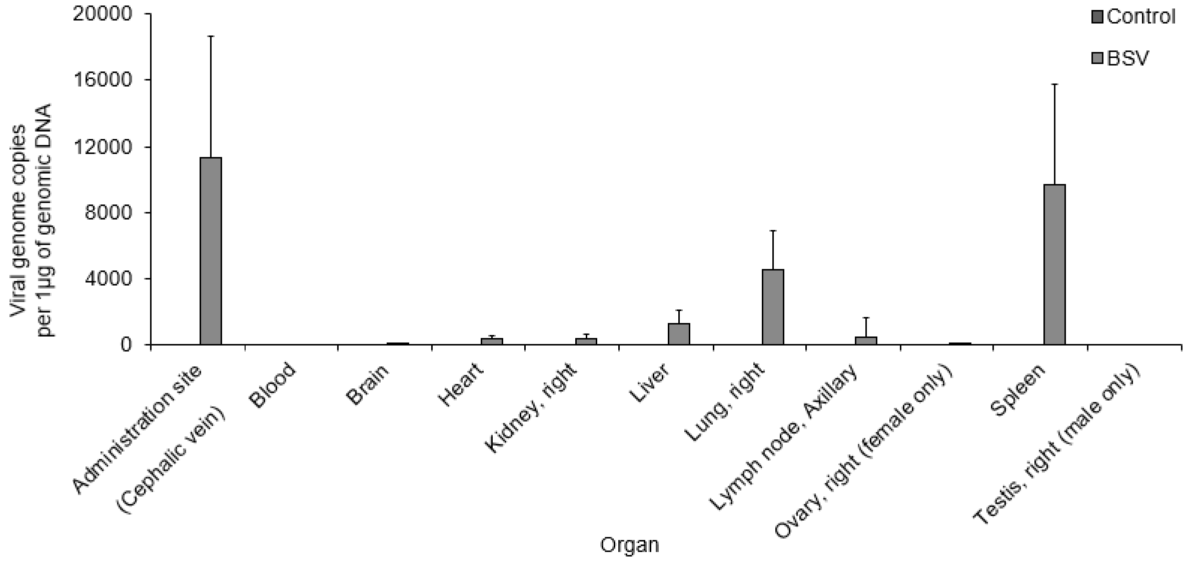

B

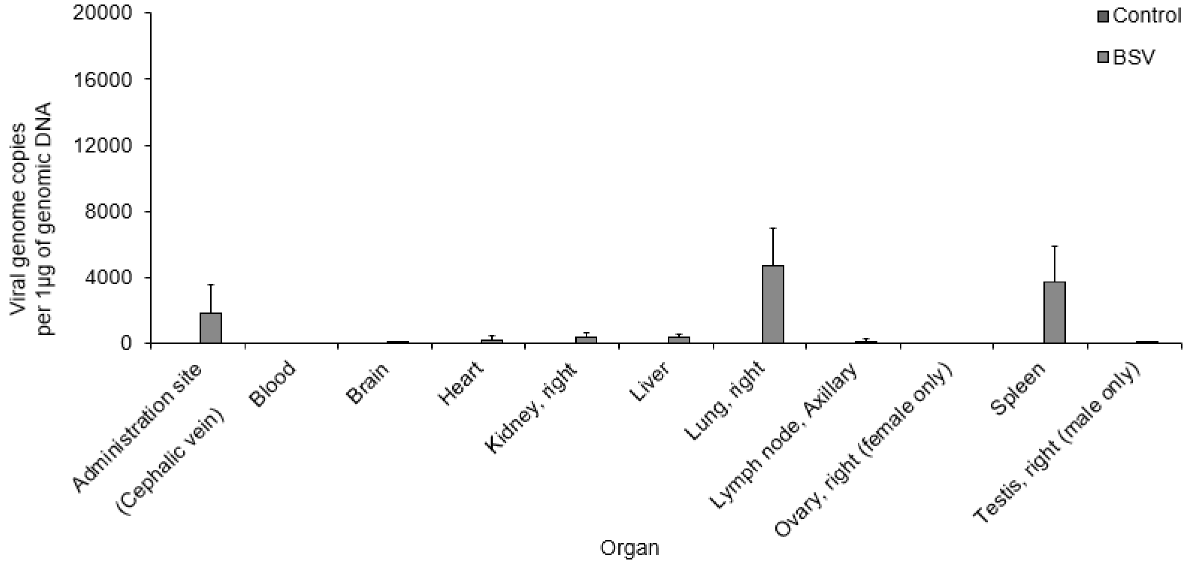

Supplementary Figure 4 Viral organ tropism as a robust method for assessing short-term (28 days) and long-term (91 days) toxicity in the hamster intravenous triple shot injection model.

In the hamster model, a multi-dose intravenous injection of 2.4×10^{10} ifu/kg BSV was administered on day 0, 7, and 14, followed by humane sacrifice of the animals at 28th (A) and 91st (B) day for viral distribution analysis in their organs. Subsequently, the genomic DNA was

extracted from each organ sample and quantified for viral particles based on the copies of viral genome. This study employed n=5 hamsters, each of which was aged 8 weeks old, for both the control and BSV-injected groups and strictly adhered to the rigorous guidelines of Good Laboratory Practice (GLP).

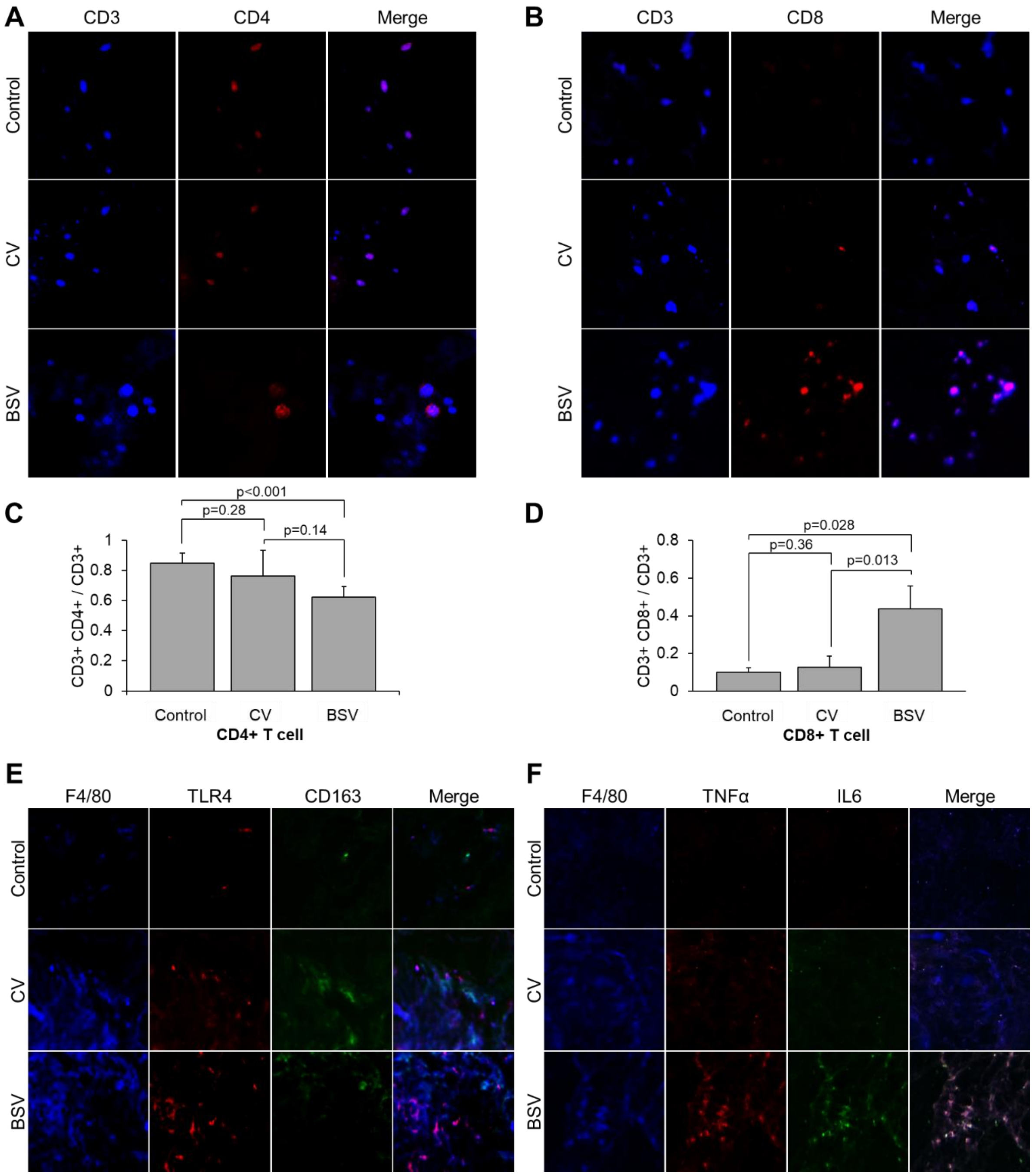

Supplementary Figure 5 T cell recruitment into tumor within xenograft mouse model

In the xenograft mouse model established by the subcutaneous injection of 1 x 10^6 253J-BV cells into 6-week-old BALB/c nude mice, T cell recruitment was analyzed via immunofluorescence staining. When the tumor volume reached 60 mm^3, 1 x 10^9 PBMCs, harvested from 6-week-old BALB/c mice, were injected intravenously into the tumor-bearing mice (n=4 per group). Concurrently, 1 x 10^8 IFU of CV and BSV were injected into the tumor.

Seven days after the PBMC and virus injections, the tumors were harvested, and the expressions of T cell markers (CD3, CD4, and CD8), macrophage markers (F4/80, TLR4 and CD163) and M1 macrophage related cytokines (TNF and IL6) were analyzed by immunofluorescence staining.

A Immunofluorescence staining to analyze CD4 positive T cell population.

B Immunofluorescence staining to analyze CD8 positive T cell population.

C Quantitative analysis of CD3 and CD4 double positive cells in CD3 positive population of (A).

D Quantitative analysis of CD3 and CD8 double positive cells in CD3 positive population of (B).

E Immunofluorescence staining to analyze TLR4 and CD163 expressions.

F Immunofluorescence staining to analyze TNFα and interleukin-6 expressions.
